# *MiR‐29a/b* Suppresses CD8^+^ T Cell Effector Function and Intestinal Inflammation

**DOI:** 10.1002/EXP.20240363

**Published:** 2025-06-10

**Authors:** Yingying Lin, Yuqi Wang, Yuning Zhang, Yao Lu, Juan Chen, Yongting Luo, Jian He, Qingfeng Luo, Heng Quan, Weiru Yu, Yujia Luo, Peng Xue, Yi Xue, Xiaoya Lin, Rui Ding, Lining Chen, Yiran Wang, Zenghui Xia, Liang Zhao, Hao Zhang, Ran Wang, Qingyu Wang, Xifan Wang, Jiaqi Su, Fazheng Ren, Cong Lv, Yixuan Li, Huiyuan Guo

**Affiliations:** ^1^ College of Food Science and Nutritional Engineering China Agricultural University Beijing China; ^2^ Key Laboratory of Functional Dairy, Department of Nutrition and Health China Agricultural University Beijing China; ^3^ Nutrition and Health Research Center National Center of Technology Innovation for Dairy Hohhot China; ^4^ Department of Gastroenterology, Beijing Hospital, National Center of Gerontology Institute of Geriatric Medicine, Chinese Academy of Medical Science Beijing China; ^5^ Department of Obstetrics and Gynecology Columbia University New York New York USA; ^6^ Department of Health Sciences and Technology ETH Zurich Zurich Switzerland

**Keywords:** CD8^+^ T cell, differentiation, *Ifng*‐JAK‐STAT, *miR‐29a/b*, ulcerative colitis

## Abstract

The role of CD8^+^ T cells in the pathogenesis of ulcerative colitis (UC) remains unclear. Similarly, the posttranscriptional regulation of the highly heterogenic CD8^+^ T cell populations and their effector function in IBD also remains poorly understood. Here, we find that *miR‐29a* and *‐29b (miR‐29a/b*) regulate T cell fate, and their expression is higher near damaged colon tissue in patients with IBD compared to controls. In mice, we find that *miR‐29a/b* suppresses the differentiation of CD8^+^ T cells and the secretion of pro‐inflammatory and chemotactic factors during severe colitis by inhibiting transcriptional pathways, including those involving the T cell receptor and JAK‐STAT signaling. Furthermore, we identify *Ifng*, an inflammatory factor that drives immune response and the reshaping of CD8^+^ T cell fate, as a potential target of the miRNAs. Finally, we show that delivery of miR‐29 mimics to the colon of mice is sufficient to alleviate DSS‐induced inflammation. Together, these data show that *miR‐29* plays an important role in suppressing T cell overactivation during inflammatory diseases.

## Graphical abstract

Our findings suggest that *miR‐29a/b* is a critical regulator of CD8^+^ T cells, possibly via the Ifng‐JAK‐STAT signal in IBD patients and the upregulation of *miR‐29a/b* relieves the development of significant inflammation in the colon of DSS‐induced colitis‐affected mice.

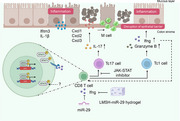

## 1 Introduction

Ulcerative colitis is a complicated and multifactorial condition marked by damage to the epithelium and the submucosal buildup of inflammatory cells [1]. Immune cells trigger vital processes during the inflammatory response. These processes include the release of proinflammatory cytokines, which enhance mucosal immunity and ultimately aid in the elimination of pathogen‐infected cells [2]. Additionally, the colonic lamina propria is home to T cells dwelling in tissue, which have the capacity to abnormally penetrate the mucosa and increase inflammation, thus contributing to tissue damage in patients with IBD [3]. Accordingly, it has been determined that aberrant T‐cell‐mediated tolerance and excessive effector T cell activation are important factors in the development and course of the illness [3]. Consequently, there is a great deal of study being done on the molecular mechanisms that control these activities.

While the majority of research has concentrated on the relationship between CD4^+^ T cells and inflammation, more focus is now being paid to the function of CD8^+^ T cells, which secrete cytokines that activate immune cells to combat pathogens during the inflammatory phase of IBD and effector molecules that kill intracellular pathogens [4]. A growing body of research indicates that the function of CD8^+^ T cells in UC has been disputed, with certain reports demonstrating its anti‐colitogenic qualities [5], while others indicate their contribution to tissue inflammation [6]. Such contradictory results could be partially explained by the origin of the CD8^+^ T cells. An additional credible rationale is based on the inherent variations of CD8^+^ T cell subsets, which exhibit a wide range of phenotypes and functions [7, 8, 9, 10]. Patients with IBD have highly heterogenic CD8^+^ T cell populations in their colons, which take on distinct phenotypes based on co‐stimulatory molecules, cytokine stimulation, and the degree of TCR‐antigen interaction [7, 8, 9]. Furthermore, studies using transcriptional profiling on peripheral CD8^+^ T cells have revealed inherent variations in patients' age, gender, and level of inflammation [11]. Additionally, memory CD8^+^ T lymphocytes that reside in the tissue of the small intestine and colon have distinct functional characteristics, in addition to specific transcriptional requirements for their upkeep [12]. Although there is evidence that CD8^+^ T cells contribute to the pathophysiology of UC, the degree of heterogeneity, transcriptional regulation, and effector function of different populations have not been thoroughly examined. Therefore, a better comprehension of the various pools of CD8^+^ T cells and the transcriptional regulation governing them may make it possible to identify therapeutic targets that will help IBD patients regain their balance and health.

MicroRNAs (miRNAs), which inhibit gene expression at the post‐transcriptional level, are changed in inflammatory bowel disease, and are crucial in controlling the disease's development [13]. In T cells, miRNAs are involved in many different biological processes and are essential for thymic development, homeostasis, survival and the activation of effector and memory cell differentiation [14]. For instance, effector cells have increased expression of *miR‐17‐92*, which promotes PI3K‐Akt‐mTOR signaling and leads to effector cell proliferation [15, 16], conversely, *miR‐155* expression promotes the growth of CD8^+^ T cells by attenuating the type I interferon's anti‐proliferative action upon infection [16]. Furthermore, *miR‐206* drives Kupffer cells' M1 polarization, which aids in the recruitment of CD8^+^ T lymphocytes [17]. All of these findings have contributed to the widespread belief that the levels of miRNAs controls the trajectory of CD8^+^ T cells, though these studies have mainly focused on their function in tumor and infection models, which are rare in IBD.

Levels of *miR‐29a* and *‐29b (miR‐29a/b*) are elevated in IBD [13], indicating that they may be involved in multiple pathophysiological networks. These miRNAs have been studied in CD4^+^ T cells [18], but minimally in the effector roles of CD8^+^ T lymphocytes in inflammatory bowel disease, though increasingly, evidence suggests that *miR‐29a/b* have a significant regulatory role in CD8^+^ T cells' memory capacity. Firstly, one of the most highly expressed miRNAs in adult mouse naïve CD8^+^ T cells is *miR‐29a* and adult mice lacking *miR‐29a* are unable to establish efficient memory recall responses during chronic infection [19]. Secondly, *miR‐29a* promotes the growth of memory‐like CD8^+^ T cells and reduces CD8^+^ T cell exhaustion during a prolonged infection [20]. Notably, possible *miR‐29a/b* targets include *Tbx21* (the gene encoding Tbet), *Eomes* and *Dnmt3a*, regulators that define CD8^+^ T cell fate [21]. In addition, other studies have reported that *miR‐29a/b* participate in the anti‐inflammatory response [22] and the regulation of helper T cell and dendritic cell fates [23, 24]. Finally, we previously found that supplementation with miR‐29b could ameliorate intestinal mucosa damage in mice given DSS (a model of colitis), as well as accelerating the recovery of damaged intestinal epithelium [25]. Thus, an important unanswered question is whether *miR‐29a/b* can participate in the differentiation process of CD8^+^ T cells in IBD to affect their effector function, in addition to regulating their the memory function.

Herein, we show that *miR‐29a/b* were significantly upregulated in patients with IBD and in DSS‐treated mice. Surprisingly, we discovered that mesenchymal intrinsic expression of *miR‐29a/b* was required to shield mice from severe DSS‐induced colitis and suppressed CD8^+^ T cell overactivation and differentiation, potentially by suppressing the Ifng‐JAK‐STAT signal. Furthermore, we developed a nanophase system to locally deliver miR‐29a/b mimics and found that approach could suppress the inflammatory response in the DSS model, suggesting the existence of a new posttranscriptional regulatory element that protects against severe intestinal inflammation when mucosal inflammatory damage occurs.

## 2 Results

### 2.1 *MiR‐29ab1* is Closely Related to the Regulation of T Cell Fate

**FIGURE 1:**
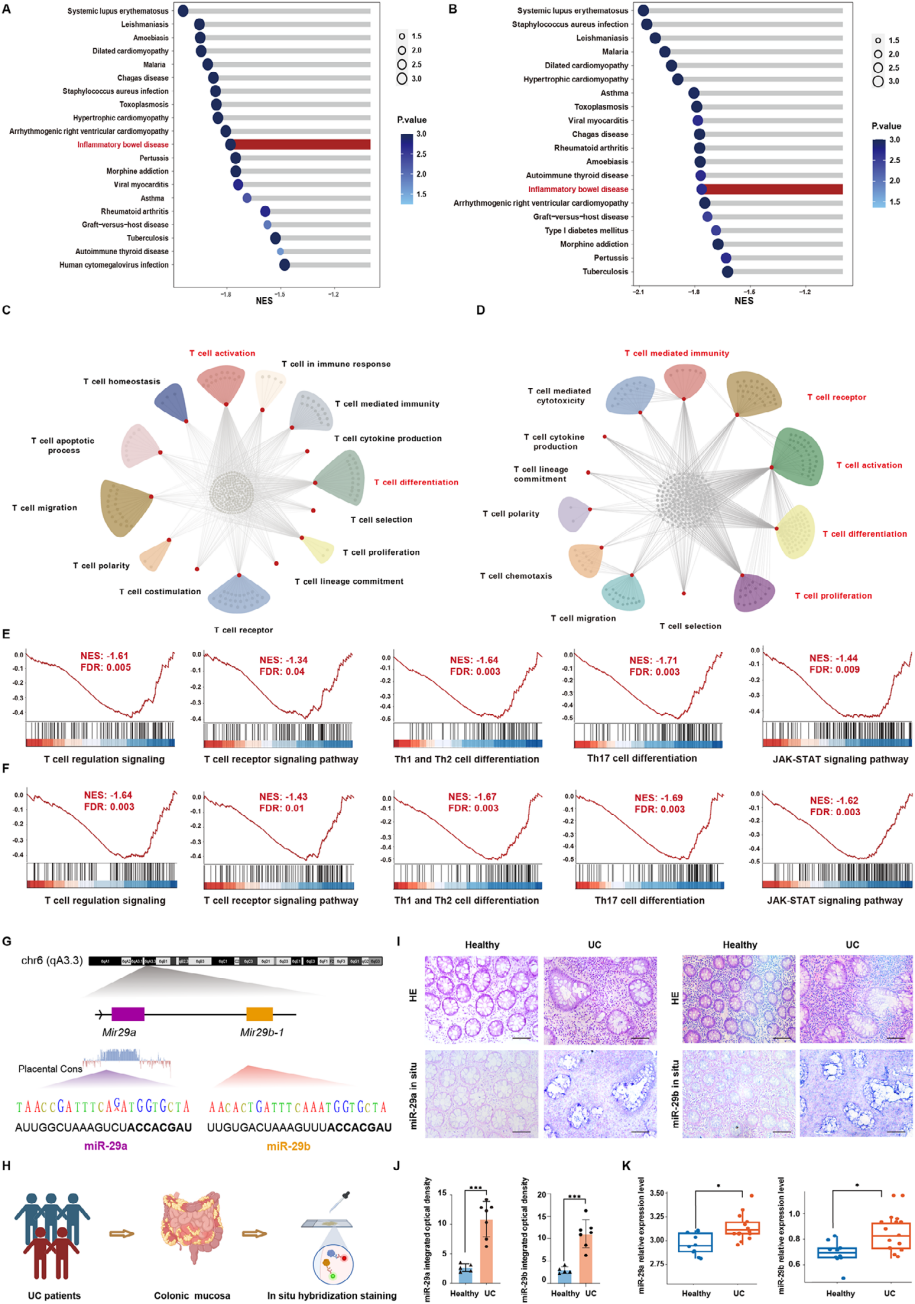
*MiR‐29a/b* is closely related to the regulation of T cell fate. (A) Disease‐related pathways enriched in GSEA on genes related to hsa‐miR‐29a‐3p in COAD and READ data set from TCGA platform. *n* = 8 normal tissues and 457 tumors in COAD, 3 normal tissues and 162 tumors in READ. (B) Disease‐related pathways enriched in GSEA on genes related to hsa‐miR‐29b‐3p in COAD and READ data set from TCGA platform. *n* = 8 normal tissues and 457 tumors in COAD, 3 normal tissues and 162 tumors in READ. (C, D) Genes related to T cell fate enriched by GO analysis. Each gray dot stands for a gene, the red dot for an enriched pathway, and the line that connects them all indicates the relationship between the genes and pathways. (E) T cell‐related pathway enriched in GSEA on genes related to hsa‐miR‐29a‐3p. The ranking metric scores (on the x‐axis) are calculated as correlation with expression of miR‐29a. (F) T cell‐related pathway enriched in GSEA on genes related to hsa‐miR‐29b‐3p. The ranking metric scores (on the *x*‐axis) are calculated as correlation with expression of miR‐29b. (G) The position and conservation of miR29a and miR29b found in UCSC GRCm38/mm10 dataset, and the sequence of miR‐29a‐3p and miR‐29b‐3p. (H) Schematic diagram showing the sample collection for in situ hybridization. (I) Histological images of colonic tissue and in situ hybridization for hsa‐miR‐29a‐3p and hsa‐miR‐29b‐3p in UC patients and healthy controls. *n* = 5–7. Scale bar: 100 µm. (J) Quantification of integrated optical density in situ hybridization for hsa‐miR‐29a‐3p and hsa‐miR‐29b‐3p in UC patients and healthy controls. *n* = 5–7. (K) The expression of hsa‐miR29a‐3p and hsa‐miR‐29b‐3p in UC and normal tissues from NCBI platform. *n* = 8–15. Data are presented as mean ± SD. Student's *t*‐test. * *p* < 0.05, ** *p* < 0.01.

Patients were ranked according to their *miR‐29a/b* expression levels and, we conducted Spearman correlation analysis between all genes of each patient and *miR‐29a/b*, and those with *p* < 0.05 were considered significantly correlated based on the TCGA‐COAD/READ dataset. GSEA was conducted based on the above genes, and all the enriched pathways associated with diseases were shown in Figure 1A,B. Inflammatory bowel disease was both negatively related to *miR‐29a* and *miR‐29b* (Figure 1A,B). In addition, the gene ontology (GO) analysis was conducted based on the above genes and we identified a large number of correlated genes related to *miR‐29a/b* belonging to T cell fate regulation processes based on the gene ontology (GO) analysis (Figure 1C,D). Furthermore, by gene set enrichment analysis (GSEA) we found that T cell differentiation, T cell receptor signaling pathway and the Janus Kinase‐signal transducer and activator of transcription (JAK‐STAT) signaling pathway, as well as numerous inflammatory signaling pathways, are negatively correlated with *miR‐29a/b* (Figure 1E,F and Figure S1A,B, Supporting Information). In view of these statistical analyses, *miR‐29a/b* was negatively related to inflammatory signaling pathways, and the miRNAs are likely to influence the inflammation response by controlling T cell fate.

Based on the negative correlation of *miR‐29a/b* with that of genes associated with the inflammatory process and T cell fate in CRC, we speculated that elevated *miR‐29a/b* also contributes to ulcerative colitis (UC) by suppressing T cell activation. Considering the absolutely high conservation of the *miR‐29a/b* locus among different species (Figure 1G), mice represent a proper model of human disease to study these miRNAs regarding their role in inflammatory responses. But first, to examine the expression change of *miR‐29a/b* in UC, we first performed in situ hybridization and found that *miR‐29a/b* was mostly expressed in the injured epithelium in patients with UC compared to adjacent healthy tissue from each of the same patients (Figure 1H–J). Furthermore, differential analysis and screening of the differential miRNA from GEO datasets (Data from GSE48959) were performed using the R language package (Limma). We discovered that *miR‐29a* and *miR‐29b* were significantly higher in the inflammatory mucosa of patients with UC patients compared to controls (Figure 1K), implying that *miR‐29a/b* is essential to response to severe colitis.

### 2.2 Deletion of *MiR‐29ab1* Contributes to T Cell Activation Under Physiological conditions

**FIGURE 2:**
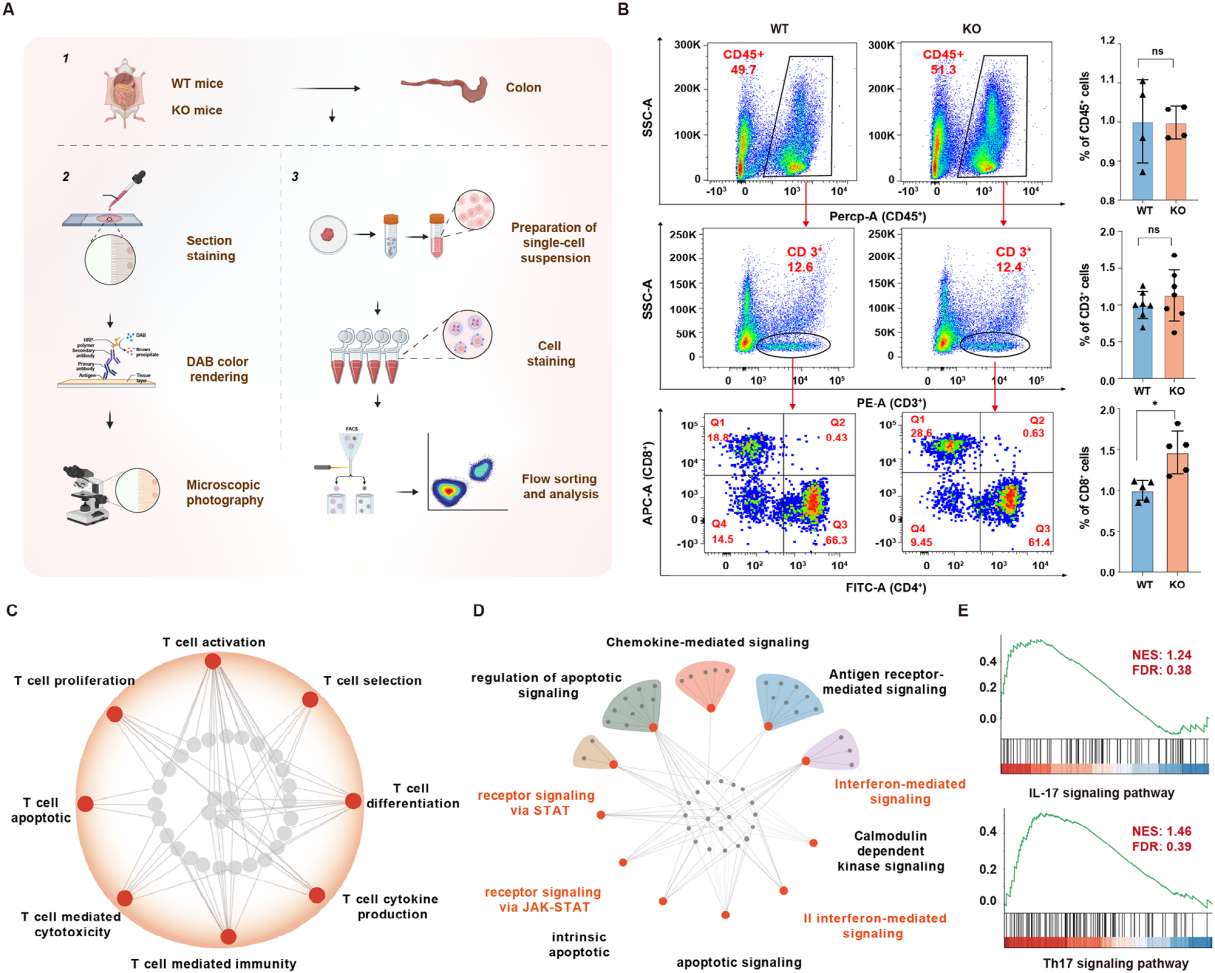
Deletion of *MiR‐29ab1* contributes to T cell activation under physiological conditions. (A) Schematic diagram showing the experimental design for detecting various types of cells in colon. (B) Flow cytometry analysis of CD45^+^, CD3^+^, and CD8^+^ cells of WT and *MiR‐29ab1^−/‐^* mice under physiological conditions. *n* = 4–7 mice in each group. (C) T cell‐related biological process enriched in GO analysis on RNA‐seq from WT and *MiR‐29ab1* ^−/‐^ mice under physiological conditions. *n* = 4 mice in each group. Each gray dot stands for a gene, the red dot for an enriched pathway, and the line that connects them all indicates the relationship between the genes and pathways. (D) Signaling pathways enriched in GO analysis on RNA‐seq from WT and *MiR‐29ab1* ^−/‐^ mice under physiological conditions. *n* = 4 mice in each group. Each gray dot stands for a gene, the red dot for an enriched pathway, and the line that connects them all indicates the relationship between the genes and pathways. (E) IL‐17 and Th17 signaling pathways enriched in GSEA from WT and *MiR‐29ab1* ^−/‐^ mice. *n* = 4 mice in each group under physiological conditions. Data are presented as mean ± SD. Student's *t*‐test or paired *t*‐test. * *p* < 0.05, ** *p* < 0.01.

Subsequently, we examined the impact of deletion of *MiR‐29ab1* on the colon by using *MiR‐29ab1* germline knockout (*MiR29ab1^−/−^_,_* KO) mice, which resulted in no significant difference in colon length and weight, crypt height, cell proliferation in the crypt or the quantity of apoptotic cells in contrast to wild‐type (WT) mice (Figure S2A–P, Supporting Information). Unexpectedly, we found that the amount of T lymphocytes with CD45^+^, CD3^+^, and CD4^+^ labels was not appreciably changed by the lack of *MiR‐29ab1*, but the quantity of CD8^+^ T lymphocytes was noticeably higher in the physiological settings than in the WT mice. (Figure 2A,B). These results imply that the lack of *MiR‐29ab1* during homeostasis modifies the landscape of colonic immune cells.

Next, we performed RNA‐sequencing (RNA‐seq) study of colon tissue from WT and *MiR‐29ab1^−/−^* mice under physiological settings to obtain mechanistic insight into how *MiR‐29ab1* contributes to the control of T cell fate. (Figure S2Q, Supporting Information). Compared to WT mice, our method revealed 305 down‐regulated and 493 up‐regulated genes in the colon tissue of *MiR‐29ab1^−/‐^* mice. (Figure S2R–T, Supporting Information). GO analysis revealed that the genes exhibiting changed expression were involved in the regulation of T cell fate, including T cell activation and differentiation (Figure 2C), and that the differentially expressed genes (DEGs) between WT and *MiR‐29ab1^−/‐^* mice contained numerous targets related to interferon‐mediated signaling and receptor signaling via JAK‐STAT, as well as the IL‐17 signaling pathway (Figure 2D–E). Together, all of these findings point to a possible function for *miR‐29a/b* in the setting of colitis.

### 2.3 Deletion of *MiR‐29ab1* Increases the Severity of DSS‐Induced Colitis by Regulating T Cell Activation and Differentiation

**FIGURE 3:**
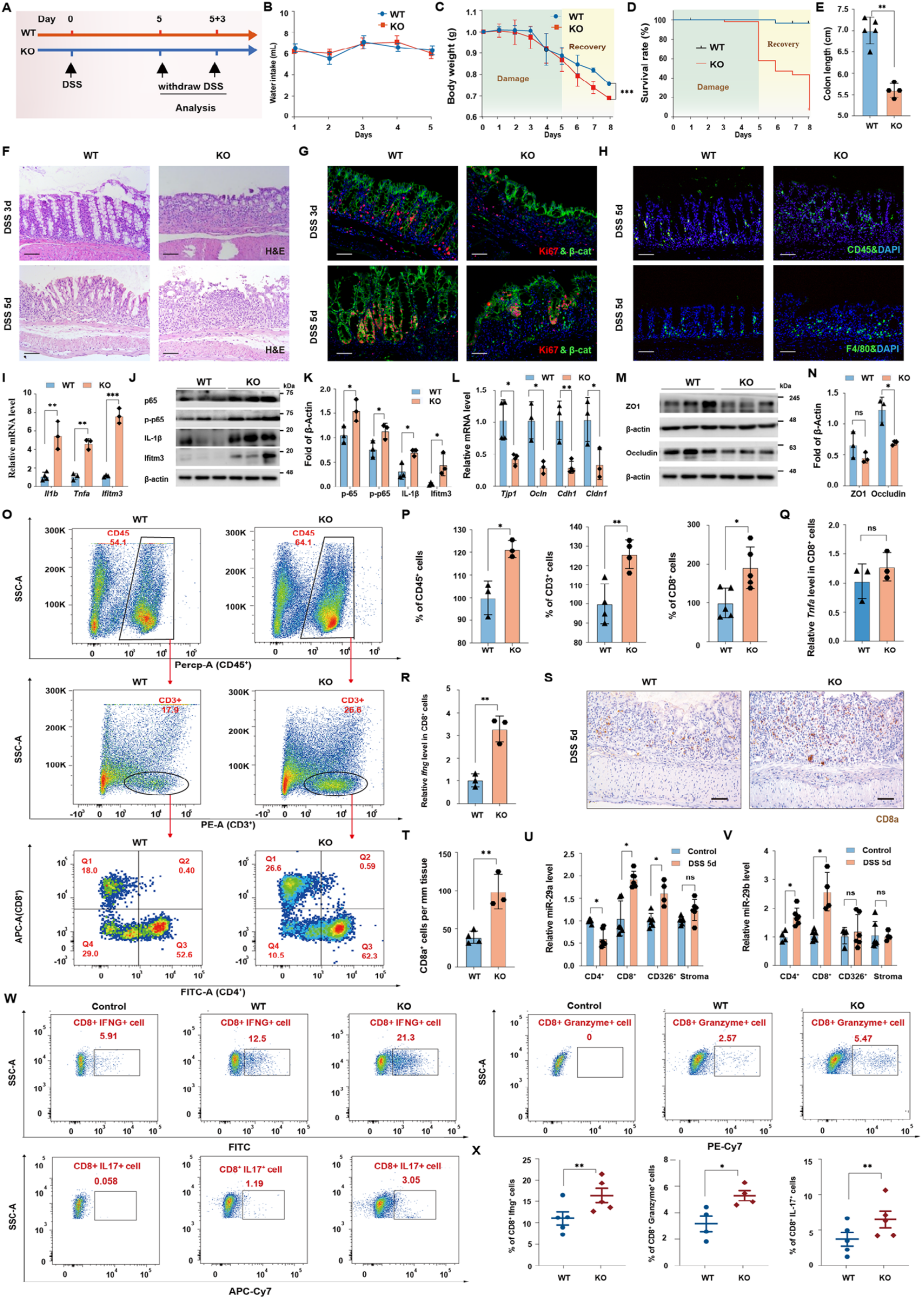
Deletion of *MiR‐29ab1* increases the severity of DSS‐induced colitis by regulating T cell activation and differentiation. (A) Schematic diagram showing DSS and analysis schedule. (B) Quantification of water intake in WT and *MiR‐29ab1^−/−^* littermates following 5 days of 3.5% DSS treatment. *n* = 6 mice in each group. (C) Quantification of weight loss in WT and *MiR‐29ab1^−/−^* littermates following 5 days of 3.5% DSS treatment. *n* = 2–18 mice in each group. (D) Survival curve of WT and *MiR‐29ab1^−/−^* littermates after 3.5% DSS treatment. *n* = 2–18 mice in each group. (E) Quantification of colon length in WT and *MiR‐29ab1^−/‐^* littermates following 5 days of 3.5% DSS treatment. *n* = 4–5 mice in each group. (F) Histological images of colonic tissue from WT and *MiR‐29ab1^−/−^* littermates at the indicated time points post DSS. Scale bar: 100 µm. *n* = 6 mice in each group. (G) Double immunofluorescence for Ki67 and β‐catenin in colon from WT and *MiR‐29ab1^−/−^* mice at the indicated time points post 3.5% DSS. Scale bar: 100 µm. n = 3 mice in each group. (H) Immunofluorescence for CD45 and F4/80 in colon from WT and *MiR‐29ab1^−/−^* mice following 5 days post 3.5% DSS. Scale bar: 100 µm. *n* = 3 mice in each group. (I) qPCR analysis for *ll1b*, *Tnfa*, and *Ifitm3* of colon from WT and *MiR‐29ab1^−/−^* littermates following 5 days post 3.5% DSS. *n* = 3–4 mice in each group. (J) Western blotting for p65, p‐p65, IL‐1β, and Ifitm3 from WT and *MiR‐29ab1^−/‐^* littermates following 5 days post 3.5% DSS. β‐actin was used as a loading control. *n* = 3 mice in each group. (K) Quantification analysis of proteins for p65, p‐p65, IL‐1β, and Ifitm3 in colon from WT and *MiR‐29ab1^−/−^* littermates following 5 days post 3.5% DSS. *n* = 3–4 mice in each group. (L) qPCR analysis for *Tjp1*, *Ocln*, *Cdh1*, and *Cldn1* of colon from WT and *MiR‐29ab1^−/−^* littermates following 5 days post 3.5% DSS. *n* = 3–4 mice in each group. (M) Western blotting for ZO1 and Occludin from WT and *MiR‐29ab1^−/−^* littermates following 5 days post 3.5% DSS. β‐actin was used as a loading control. *n* = 3 mice in each group. (N) Quantification analysis of proteins for ZO1 and Occludin from WT and *MiR‐29ab1^−/−^* littermates following 5 days post 3.5% DSS. *n* = 3 mice in each group. (O) Flow cytometry analysis of CD45^+^ T leukocytes, CD3^+^ cells and CD4^+^ cells in the colon from WT and *MiR‐29ab1^−/−^* littermates following 3 days of 3.5% DSS treatment. *n* = 6 mice in each group. (P) Quantification of CD45^+^ T leukocytes, CD3^+^ cells and CD8^+^ cells in the colon from WT and *MiR‐29ab1^−/−^* littermates following 3 days of 3.5% DSS treatment. *n* = 3–5 mice in each group. (Q, R) qPCR analysis for *Tnfa* and *Ifng* level in CD8^+^ cells from WT and *MiR‐29ab1^−/−^* littermates following 3 days post 3.5% DSS. *n* = 3 mice in each group. (S–T) Immunofluorescence images and quantification of CD8a^+^ cells in the colon from WT and *MiR‐29ab1^−/−^* littermates following 5 days post 3.5% DSS. Scale bar: 100 µm. *n* = 3–4 mice in each group. (U–V) qPCR analysis for relative *miR‐29a* and *miR‐29b* level in CD4^+^, CD8^+^, CD326^+^, and stroma cells from WT mice following 5 days post 3.5% DSS. *n* = 4–6 mice in each group. (W–X) Flow cytometry analysis and quantification for the percentage of Ifng^+^ CD8^+^ cells, Granzyme^+^ CD8^+^ cells, and IL‐17^+^ CD8^+^ cells in the colon from WT and *MiR‐29ab1^−/−^* littermates following 5 days post 3.5% DSS. Data are presented as mean ± SD. *n* = 4–6 mice in each group. Data are presented as mean ± SD, except that data in (X) are presented as mean ± SEM. Student's *t*‐test. * *p* < 0.05, ** *p* < 0.01.

As CD8^+^ T cells were abnormally activated by *MiR‐29ab1* deletion, we hypothesized that such alterations were magnified in the context of inflammation, driving the exacerbation of UC. To gain insight into the function of *MiR‐29ab1* in colitis, we used DSS in the drinking water for 5 days to cause colitis in both WT and *MiR‐29ab1^−/−^* mice. After that, the mice recovered for 3 days by drinking untreated water (Figure 3A). To guarantee DSS intake, drinking water containing DSS was also regularly checked, with no difference in overall water intake between WT and mutant mice (Figure 3B). Following 5 days of DSS treatment, *MiR‐29ab1^−/−^* mice lost much more weight than WT mice treated with DSS on the 7th day, and their survival rate was also lower (Figure 3C,D). Histologically, *MiR‐29ab1^−/−^* mice had a shorter colon length and marked colon epithelial damage by day 5 compared to similarly treated WT mice (Figure 3E,F), with delayed intestinal regeneration by day 8 (Figure S3A, Supporting Information). A time‐course analysis revealed markedly less epithelial cell proliferation in *MiR‐29ab1^−/−^* mice in contrast to WT mice (Figure 3G and Figure S3B, Supporting Information). Furthermore, we found evidence of less barrier function in the colonic epithelium of *MiR‐29ab1^−/−^* mice, massive infiltration of myeloid cells, including leukocytes and macrophages, and higher levels of proinflammatory cytokines in *MiR‐29ab1^−/−^* mice in contrast to WT mice after DSS treatment (Figure 3H–N and Figure  S3C, Supporting Information).

To explore the physiological function of *MiR‐29ab1* in T cell responses, we analyzed T cell numbers by flow cytometry. Following DSS therapy, we described the heightened immunological response in *MiR‐29ab1^−/−^* mice and discovered that the loss of *MiR‐29ab1* resulted in a markedly greater number of CD8^+^ T lymphocytes, along with CD3^+^ and CD45^+^ T lymphocytes, and higher levels of the proinflammatory cytokine IFN‐γ from CD8^+^ T cells compared to DSS‐treated WT mice after 3 days of therapy (Figure 3O–R). These results were further confirmed by a higher CD8^+^ T cell count in the mutant mice as opposed to WT mice given DSS (Figure 3S,T). In concordance with the critical function of *MiR‐29ab1* in CD8^+^ T lymphocytes, *MiR‐29ab1* revealed notably greater numbers in CD8^+^ T cells from WT mice treated with DSS as opposed to WT animals treated with control (Figure 3U,V), indicating the locus likely participates in the regulation of T cells.

Through the secretion of several inflammatory factors, CD8^+^ T lymphocytes have a well‐established role in controlling inflammatory processes in various tissue types. To further assess CD8^+^ T lymphocytes effector function, we employed flow cytometry to analyze T cell numbers and found the percentage of IFN‐γ^+^ CD8^+^ T lymphocytes (additionally known as type 1 CD8^+^ T lymphocytes (Tc1 cells), Granzyme^+^ CD8^+^ T lymphocytes and IL‐17^+^ CD8^+^ T lymphocytes (additionally known as Tc17 cells) were greater in the mutant mice than in WT mice (Figure 3W,X), indicating enhanced T cell differentiation. Together, our data show that via controlling T cell activation and differentiation, *MiR‐29ab1* is essential in reducing the inflammatory response to DSS therapy.

### 2.4 Deletion of *MiR‐29ab1* Activates the JAK‐STAT and IL‐17 Signaling Pathways in Colonic Stromal Cells

**FIGURE 4:**
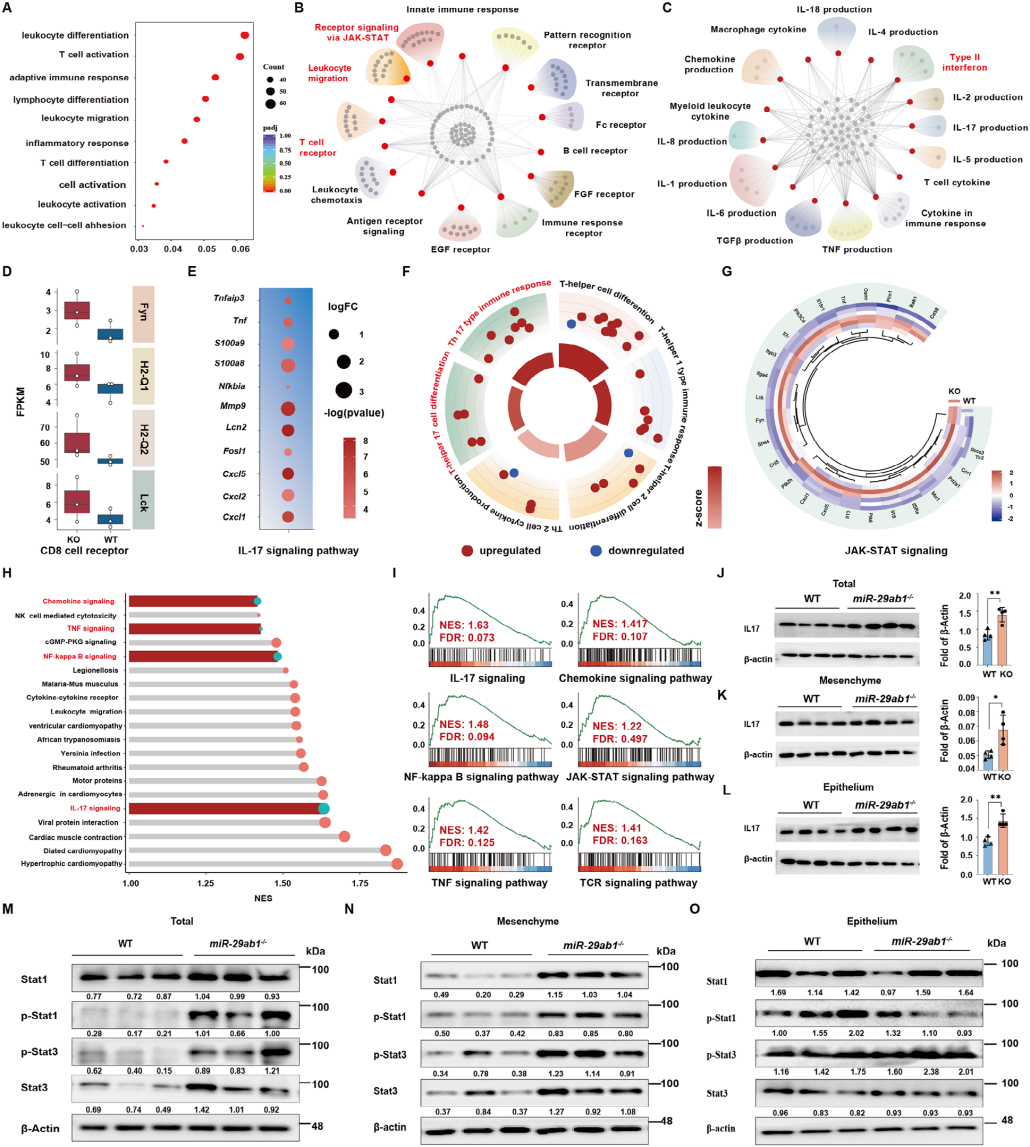
Deletion of *MiR‐29ab1* activates the JAK‐STAT and IL‐17 signaling pathways in colonic stromal cells. (A) Top 10 biological process enriched in GO. *n* = 3 mice from WT and *MiR‐29ab1* ^−/‐^ mice followed by 3 days 3.5% DSS treatment. (B, C) GO analysis of RNA‐sequencing data from WT and *MiR‐29ab1* ^−/‐^ mice followed by 3 days 3.5% DSS treatment. Red points represent the most significant biological processes. Grey points represent the genes belonging to these processes. *n* = 3 mice in each group. (D) Genes regulating CD8 cell receptors in WT and *MiR‐29ab1* ^−/‐^ mice followed by 3 days 3.5% DSS treatment. *n* = 3 mice in each group. (E) Changes in genes involved in IL‐17 signaling pathway in *MiR‐29ab1* ^−/‐^ compared with WT mice followed by 3 days 3.5% DSS treatment. *n* = 3 mice in each group. (F) Changes in genes related to T helper cells in *MiR‐29ab1* ^−/−^ compared with WT mice followed by 3 days 3.5% DSS treatment. (G) Heatmap of differential genes expression analysis related to JAK‐STAT signaling followed by 3 days 3.5% DSS treatment. *n* = 3 mice in each group. (H) GSEA analysis of top 20 biological process from WT and *MiR‐29ab1* ^−/‐^ mice followed by 3 days 3.5% DSS treatment. *n* = 3 mice in each group. (I) GSEA analysis of colonic tissue from WT and *MiR‐29ab1* ^−/‐^ mice followed by 3 days 3.5% DSS treatment. *n* = 3 mice in each group. (J–L) Western blotting and quantification for IL‐17 in the entire colon, and in the mesenchyme and epithelium of colon from WT and *MiR‐29ab1* ^−/‐^ mice followed by 3 days 3.5% DSS treatment, respectively. β‐actin was used as a loading control. *n* = 4 mice in each group. (M–O) Western blotting and quantification for Stat1, p‐Stat1, p‐Stat3, Stat3 in the entire colon, the mesenchyme and epithelium of colon from WT and *MiR‐29ab1* ^−/‐^ mice followed by 3 days 3.5% DSS treatment, respectively. β‐actin was used as a loading control. *n* = 3 mice in each group. Data are presented as mean ± SD. Student's *t*‐test. * *p* < 0.05, ** *p* < 0.01.

We performed an RNA‐seq analysis on colonic tissue from WT and *MiR‐29ab1^−/−^* mice following 3 days of DSS therapy in order to obtain mechanistic insight into how *MiR‐29ab1* contributes to the regulation of CD8^+^ T lymphocytes differentiation. The tissue from the *MiR‐29ab1^−/−^* mice showed 531 down‐regulated genes and 753 up‐regulated genes in comparison to the WT controls. (Figure S4A–C, Supporting Information). By GO analysis, we found that the genes with altered expression were involved in T cell fate regulation, including T cell activation and adaptive immune response, especially T cell differentiation (Figure 4A). Further, we found that the DEGs between WT and *MiR‐29ab1^−/−^* mice contained numerous targets related to interferon‐mediated signaling and receptor signaling via the JAK‐STAT, as well as the IL‐17, signaling pathway (Figure 4B,C, Figure S4D, Supporting Information), which was consistent with the RNA‐seq data from physiological conditions described above (Figure 2C–E). The expression of genes related to CD8^+^ T cell receptors, the IL‐17 signaling pathway, T‐helper 17 cell differentiation and Th17 type immune response and the JAK‐STAT signaling pathway were higher in the colon tissue from *MiR‐29ab1^−/−^* mice compared to WT mice followed 3 days of DSS treatment (Figure 4D–G). In accordance with the TCGA database, a large number of the correlated genes are related to T cell fate regulation based on the gene ontology (GO) analysis (Figure S4E,F, Supporting Information). Furthermore, by GSEA analysis we found an association between *miR‐29a/b* and a large number of inflammation signaling pathways, especially the IL‐17 signaling pathway (Figure 4H,I).

In order to clarify the fundamental connection between *miR‐29a/b* and the aforementioned signaling pathways, we first focused on the IL‐17 expression level after DSS treatment and found higher expression in *MiR‐29ab1^−/−^* mice compared to WT mice (Figure 4J–L), indicating an inverse correlation between IL‐17 and *MiR‐29ab1*. Apart from the IL‐17 signaling, we also paid attention to the JAK‐STAT signaling pathway based on the RNA‐seq results, which is the other pathway important for the function of various innate and adaptive cell types that support colonic inflammation. When compared to similarly treated WT mice, we discovered that the colons of *MiR‐29ab1^−/−^* mice had considerably greater expression levels of Stat1, p‐Stat1, Stat3, and p‐Stat3 following DSS therapy (Figure 4M). In addition, we found the JAK‐STAT signal was activated in the colonic mesenchyme in the mutant mice in contrast to the WT mice, whereas there was no discernible change in expression of these proteins in the colonic epithelium (Figure 4N,O, Figure S4G, Supporting Information), suggesting a direct effect of *MiR‐29ab1* on JAK‐STAT activity in the colonic mesenchyme. The results were confirmed by the epithelium‐specific knockout of *MiR‐29ab1* via the use of *Vil*‐Cre, which showed no aggravation of inflammatory injury compared to the WT controls (Figure S5A–L, Supporting Information). In summary, *miR‐29a/b* likely participates in inflammation reactions by regulating the fate of the IL‐17 and JAK‐STAT pathways.

### 2.5 *MiR‐29a/b* Regulates CD8^+^ T Cell Activation and Differentiation Through an IFN‐γ‐JAK‐STAT Signaling Pathway

**FIGURE 5:**
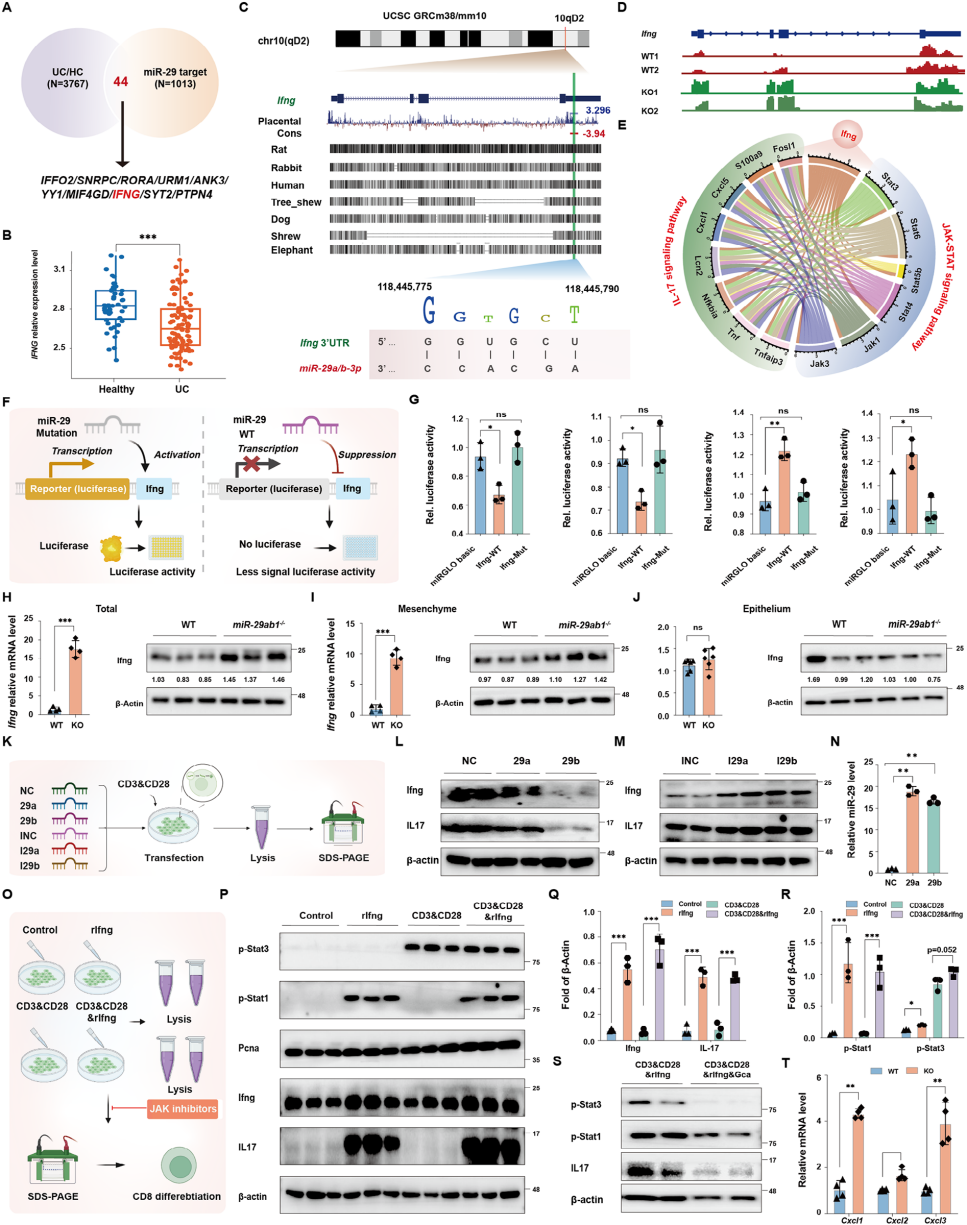
*MiR‐29a/b* regulates CD8^+^ T cell activation and differentiation through an Ifng‐JAK‐STAT signaling pathway. (A) Intersection of the *miR‐29a/b* target gene database and genes with significant changes in UC patients. (B) The expression of *IFNG* in UC and normal tissues from NCBI platform. *n* = 37 normal tissues and 70 inflammatory tissues from IBD patients. (C) Conservation of the binding cite with *miR‐29a/b* in *Ifng*. (D) The track of *Ifng* detected in RNA seq on WT and *MiR‐29ab1* ^−/−^ mice followed by 3 days 3.5% DSS treatment. (E) The correlation determined by spearman method between *Ifng*, genes related to the IL‐17 signaling pathways (located on the green ribbon) and JAK‐STAT signaling pathways (located on the blue ribbon). Various colors correspond to different genes, the more genes there are, while the stronger the correlation between pathways. (F) Schematic diagram showing the design for the luciferase experiment. (G) Luciferase activity in 293T cells treated with 40 nm miR‐29a/b mimics and negative control (NC, Scramble RNA), as well as miR‐29a/b inhibitor and negative control (NC, Scramble RNA) for 24 h. *n* = 3 independent experiments. (H–J) qPCR analysis and western blotting for Ifng in the entire colon, the mesenchyme and epithelium of colon from WT and *MiR‐29ab1* ^−/‐^ mice followed by 5 days 3.5% DSS treatment, respectively. β‐actin was used as a loading control. *n* = 4–6 mice in each group. (K) Schematic diagram showing the design for the cell experiment verifying the effect of 40 nm miR‐29a/b on CD8^+^ cells. (NC, negative control of mimics; 29a, miR‐29a mimics; 29b, miR‐29b mimics; INC, negative control of inhibitor; I29a, miR‐29a inhibitor; I29b, miR‐29b inhibitor). (L) Western blotting for Ifng and IL‐17 after 48 h of the transfection of 40 nm miR‐29a/b mimics. β‐actin was used as a loading control. *n* = 3 independent experiments. (M) Western blotting for Ifng and IL‐17 after 48 h of the transfection of miR‐29a/b inhibitor. β‐actin was used as a loading control. *n* = 3 independent experiments. (N) qPCR analysis for the relative expression of *miR‐29a/b* after 24 h treated with 40 nm miR‐29a/b mimics and negative control (NC, Scramble RNA). *n* = 3 independent experiments. (O) Schematic diagram showing the design for whether Ifng is responsible for the modulation of CD8^+^ cell differentiation. (P) Western blotting for p‐Stat3, p‐Stat1, Pcna, Ifng and IL‐17 in CTLL‐2 cells treated with or without rIfng for 48 h. β‐actin was used as a loading control. *n* = 3 independent experiments. (Q, R) Quantification analysis of proteins of Ifng, IL‐17, p‐Stat1, and p‐Stat3 treated with or without rIfng for 48 h. *n* = 3 independent experiments. (S) Western blotting for p‐Stat3, p‐Stat1, and IL‐17 in CTLL‐2 cells treated with rIfng or rIfng and Ganoderic acid A for 48 h. β‐actin was used as a loading control. *n* = 3 independent experiments. (T) qPCR analysis for the expression of *Cxcl1, Cxcl2*, and *Cxcl3* in colonic epithelium from WT and *MiR‐29ab1^−/^* mice followed by 5 days 3.5% DSS treatment. *n* = 4 mice in each group. Data are presented as mean ± SD. Student's *t*‐test (H, I, J, T) or Duncan's post hoc test following one‐way ANOVA (G, N, Q, R). * *p* < 0.05, ** *p* < 0.01.

Reduction of mRNA stability is a major way by which miRNAs suppress the expression of genes. Therefore, we used Target Scan algorithms (https://www.targetscan.org/mamm_31/) to examine *miR‐29a/b* binding locations in 3' untranslated regions (UTRs) of transcripts encoding proteins important in the intestinal immune response. To specifically seek out the targets of *miR‐29a/b* that are participating in the reprogramming of inflammatory responses and CD8^+^ T lymphocyte differentiation, we took the intersection between *miR‐29a/b* target genes from the Target Scan database and significantly downregulated genes in patients with UC (Datas from GSE119600). By this manner we found that *IFNG* is the only gene that contains a predictable *miR‐29a/b* consensus site in the 3'‐UTR among all the DEGs related to inflammation, and the binding sites are conserved among all the species (Figure 5A–C). Then, differential analysis for IFNG from GEO datasets (Datas from GSE119600) was performed using the R language package (Limma). Analysis results showed that *IFNG* is lower in patients with UC compared to their healthy adjacent tissue, contrary to *miR‐29a/b* expression (Figure 5B). Similarly, the results showed that *Ifng* was highly expressed in the colon tissue of *MiR‐29ab1^−/−^* mice compared with WT mice following 3 days of DSS treatment (Figure 5D). Gene network interaction analysis further revealed that a regulatory effect of the *Ifng* and the JAK‐STAT signaling pathway on the IL‐17 signaling pathway (Figure 5E). Furthermore, by 3′ UTR‐luciferase reporter assays we discovered that a miR‐29a/b mimic strikingly suppressed WT *Ifng* 3′ UTR activity but had no significant effect on reporters with the *miR‐29a/b* binding site mutated. In contrast, miR‐29a and miR‐29b inhibitors strikingly increased WT *Ifng* 3′ UTR activity but had no effect on reporters with a mutated *miR‐29a/b* binding site (Figure 5F,G). In agreement, Ifng was significantly higher in the whole colon and colonic mesenchyme of *MiR‐29ab1^−/−^* mice following 5 days of DSS therapy, but not in the colonic epithelium in contrast to WT mice (Figure 5H–J), indicating that *miR‐29a/b* modulates Ifng expression, especially in the colonic mesenchyme, rather than the colonic epithelium.

Next, we performed the following experiments to confirm the impact of *miR‐29a/b* regulation on CD8^+^ T cells (Figure 5K). We discovered a higher expression of IL‐17 and Ifng when *miR‐29a/b* was inhibited in CTLL‐2 cells, and conversely, lower expression when *miR‐29a/b* was overexpressed in CTLL‐2 cells, compared to controls (Figure 5L–N). These results imply a possible role of Ifng in miR‐29a/b‐mediated control of CD8^+^ T lymphocyte activity throughout the inflammatory response.

To determine whether Ifng is responsible for the modulation of CD8^+^ T cell differentiation, we treated CTLL‐2 cells with 200 ng/mL of mouse recombinant Ifng (rIfng) for 48 h (Figure 5O), which resulted in higher levels of Pcna under the joint stimulation with mouse recombinant CD3 and CD28 and increased endogenous Ifng, IL‐17 and p‐Stat1. However, the expression of p‐Stat3 was not dependent on rIfng treatment, but rather on CD3 and CD28 co‐treatment, with no effect by rIfng treatment (Figure 5P–R). Meanwhile, the induction of these proteins after rIfng treatment was suppressed upon JAK‐STAT inhibition (Ganoderic acid A, Gca) (Figure 5S, Supporting Information). Meantime, our results demonstrated that KO mice treated with static had a longer colon length and less colon epithelial damage by day 5 compared to similarly treated KO mice, indicating the important regulatory role of *miR‐29a/b* in the JAK‐STAT1/3 pathway in UC (Figure S6A–D, Supporting Information). Moreover, we also found the mRNA expression of important chemokines (*Cxcl1, Cxcl2*, and *Cxcl3*) in colonic epithelium was significantly higher in *MiR‐29ab1^−/−^* mice in contrast to WT mice (Figure 5T). Overall, these data show that the function of *miR‐29a/b* in CD8^+^ T cell differentiation is dependent on the Ifng‐JAK‐STAT signaling pathway.

### 2.6 A Hydrogel Delivery System of a miR‐29a/b Mimic is Sufficient to Alleviate DSS‐Induced Inflammation

**FIGURE 6:**
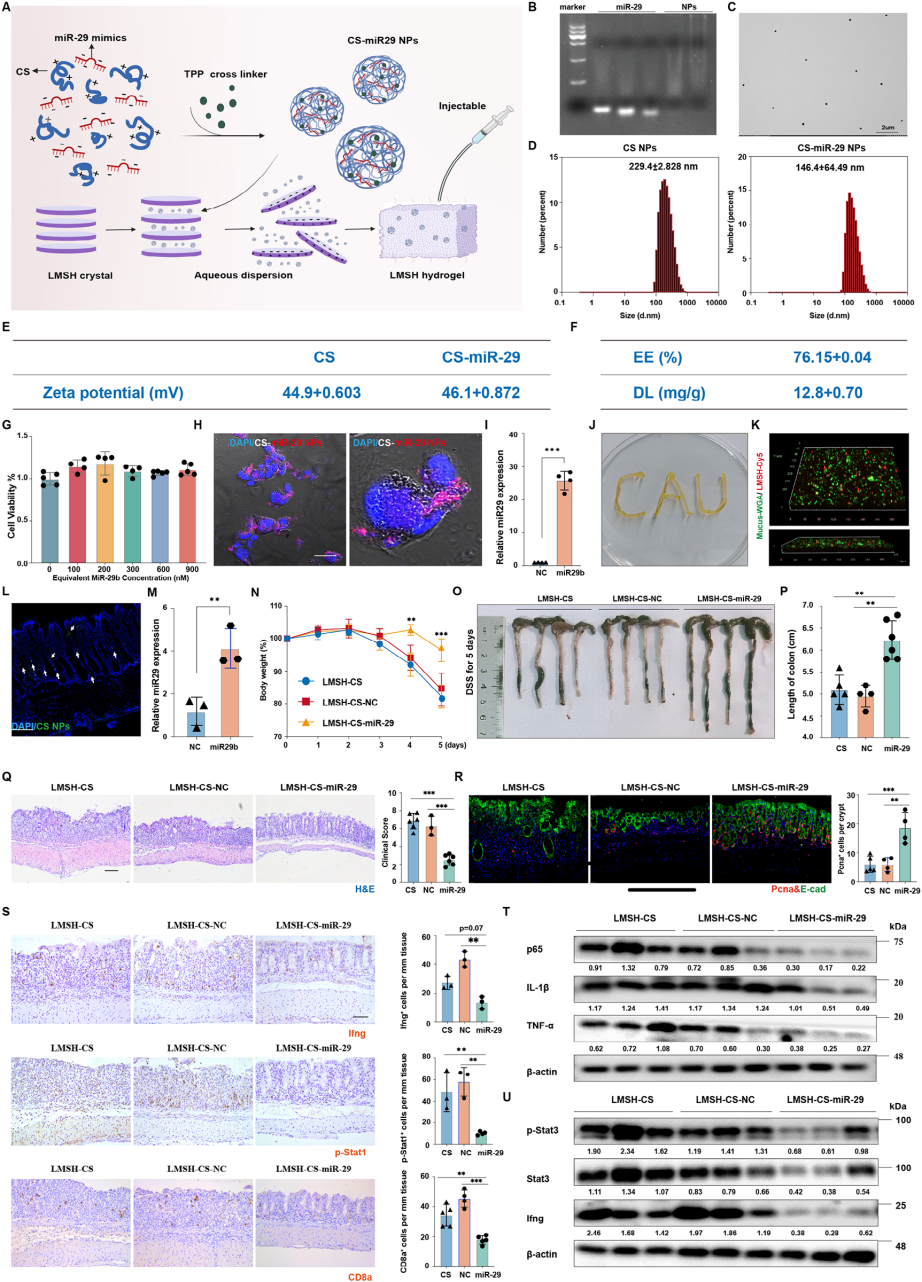
A hydrogel delivery system of a miR‐29a/b mimic is sufficient to alleviate DSS‐induced inflammation. (A) Schematic diagram showing the design and synthesis strategy of the LMSH‐NPs‐ miR‐29a/b hydrogel. (B) Encapsulation of miR‐29a/b mimics loading to CS NPs. *n* = 3 technical replicates. (C) Transmission electron microscopy images of CS‐miR‐29a/b NPs. *n* = 3 technical replicates. (D) Size distribution of CS‐ miR‐29a/b NPs by intensity. *n* = 3 technical replicates. (E) Zeta potential of CS NPs and CS‐miR‐29 NPs. *n* = 3 technical replicates. (F) The encapsulation efficiency (EE) of CS‐ miR‐29a/b NPs. *n* = 3 technical replicates. (G) CCK‐8 analysis of the viability of CaCO_2_ cells treated with CS‐miR‐29 NPs at indicated concentrations after 24 h of incubation. *n* = 3 technical replicates. (H) Confocal microscopy images showing the internalization of the miR‐29a/b mimics (labeled by Cy5) by CaCO_2_ cells delivered with NPs 6 h after treatment. *n* = 3 technical replicates. (I) qPCR analysis for relative *miR‐29a/b* expression in CaCO_2_ cells delivered with NPs 6 h after treatment. *n* = 4 mice in each group. (J) Representative picture showing the injectability of the LMSH‐NPs‐ miR‐29a/b hydrogel. (K) CLSM images of LMSH‐cy5 hydrogel (red) in colon mucosal surfaces layer (green) after 1 h of rectal administration. Scale bar: 50 µm. *n* = 3 technical replicates. (L) Fluorescence microscopy images showing the intake of miR‐29a/b by epithelial cells in the colon. The mice were treated with LMSH‐NPs‐ miR‐29a/b hydrogel by enema for 6 h. Scale bar: 50 µm. *n* = 3 technical replicates. (M) qPCR analysis for relative *miR‐29a/b* expression in the epithelial of colonic tissues for 3 days of LMSH‐NPs‐miR‐29a/b hydrogel treatment by enema. *n* = 3 mice in each group. (N) Quantification of weight loss in WT mice following 5 days of DSS treatment, coupled with LMSH‐NPs, LMSH‐NPs‐NC, LMSH‐NPs‐ miR‐29a/b treatment. *n* = 4–6 mice in each group. (O) Representative gross images of the colon of mice treated with LMSH‐NPs, LMSH‐NPs‐NC, LMSH‐NPs‐ miR‐29a/b treatment followed by 5 days 3.5% DSS treatment. *n* = 4–6 mice in each group. (P) Quantification of colon length under the different condition in (O). *n* = 4–6 mice in each group. (Q) Hematoxylin‐eosin (HE) staining and quantification of the clinical scores in the colon from WT mice following 5 days of DSS treatment, and treated with LMSH‐NPs, LMSH‐NPs‐NC, LMSH‐NPs‐ miR‐29a/b hydrogel. Scale bar: 100 µm. *n* = 3–6 mice in each group. (R) Double immunofluorescence for Pcna and E‐cad and quantification of Pcna^+^ cells per crypt in the colon from WT mice following 5 days of DSS treatment, and treated with LMSH‐NPs, LMSH‐NPs‐NC, LMSH‐NPs‐miR‐29a/b hydrogel. Scale bar: 50 µm. *n* = 4–6 mice in each group. (S) Immunohistochemistry and quantification for Ifng^+^, p‐Stat1^+^, and CD8a^+^ cells in the colon from WT mice following 5 days of DSS treatment, and treated with LMSH‐NPs, LMSH‐NPs‐NC, LMSH‐NPs‐miR‐29a/b hydrogel. Scale bar: 50 µm. *n* = 3–6 mice in each group. (T) Western blotting for p65, IL‐1β, TNF‐α in the colon from WT mice following 5 days of DSS treatment, and treated with LMSH‐NPs, LMSH‐NPs‐NC, LMSH‐NPs‐ miR‐29a/b hydrogel. β‐actin was used as a loading control. *n* = 4–6 mice in each group. (U) Western blotting for p‐Stat3, Stat3, and Ifng in the colon from WT mice following 5 days of DSS treatment, and treated with LMSH‐NPs, LMSH‐NPs‐NC, LMSH‐NPs‐miR‐29 hydrogel. β‐actin was used as a loading control. *n* = 4–6 mice in each group. Data are presented as mean ± SD. The ANOVA test (I, M) or Duncan's post hoc test following one‐way ANOVA (G, N, P, Q, R, S). * *p* < 0.05, ** *p* < 0.01.

Therapeutic approaches for IBD utilizing hydrogel delivery methods based on microRNA have attracted a lot of interest. To determine the feasibility of an IBD treatment based on *miR‐29a/b*, we designed a miR‐29a/b mimic‐based hydrogel delivery system (Figure 6A). In this system, miR‐29a/b mimics were loaded into chitosan (CS) nanoparticles in the presence of the ionic crosslinker tripolyphosphate (TPP) based on the mechanism of electrostatic interaction, which can self‐assemble into roughly 200‐nm nanoparticles (NPs) with a positive Zeta potential (Figure 6B–E). A gel mobility assay further confirmed the encapsulation of miR‐29a/b mimics, as the chitosan‐ miR‐29a/b mimic nanoparticles (CS‐miR‐29 NPs) did not migrate towards the positive electrode as the unencapsulated miR‐29a/b mimics (Figure 6B). The encapsulation efficiency (EE) of CS‐ miR‐29a/b NPs was 76%, and approximately 12 mg of miR‐29a/b mimics were loaded in CS NPs per gram (Figure 6F). The CS‐miR‐29 NPs exhibited low cytotoxicity (Figure 6G, Figure S7A–D, Supporting Information) and were capable of delivering miR‐29a/b into cells (Figure 6H). We enclosed the NPs‐miR‐29a/b nanoparticles inside a lithium magnesium silicate hydrogel (LMSH), which is created by the mutual attraction of positive and negative charges between the two, in order to prevent the nanoparticles from degrading in the gut. (Figure 6A). The LMSH‐CS‐miR‐29a/b hydrogel was then effectively administered by enema (Figure 6I) to examine their therapeutic effect in vivo. We found that treatment with the LMSH‐NPs‐miR‐29a/b hydrogel could adhere to the intestinal mucus and release NPs‐miR‐29a/b into colonic epithelial cells, leading to an increase in miR‐29a/b levels in the colon (Figure 6J,K).

We ultimately tested whether the LMSH‐NPs‐ miR‐29a/b hydrogel could alleviate DSS‐induced inflammation. We continuously administered LMSH‐NPs hydrogel to the large bowel of WT mice, and 3 days later we concurrently fed the mice drinking water with 3.5% DSS for 5 days. Compared with the treatment with LMSH‐NPs negative controls, treatment with LMSH‐NPs‐miR‐29a/b hydrogel ameliorated body weight loss and colon shortening (Figure 6N–P). Strikingly, the LMSH‐NPs‐miR‐29a/b hydrogel reduced inflammation and compromised integrity of the colon's epithelium as indicated by a lower clinical score and greater epithelial proliferation (Figure 6Q–S). Consistent with our findings above, the JAK‐STAT signaling pathway was significantly lower in the colon of LMSH‐NPs‐miR‐29a/b hydrogel‐treated mice, whereas Ifn‐γ levels (with its mRNA being the target of miR‐29a/b) was lower, in contrast to control‐treated mice (Figure 6T,U). These findings indicated that LMSH‐NPs‐ miR‐29a/b hydrogel is efficient in treating mice with active colitis. Collectively, our results indicate that LMSH‐NPs‐ miR‐29a/b hydrogel may be a powerful treatment strategy for IBD.

## 3 Discussion

In this article, we identified the critical roles of Tc1 and Tc17 cells in UC and showed how *miR‐29* regulates them through JAK‐STAT signal pathway. We showed that *miR‐29a/b* expression was dramatically elevated in the inflamed colon tissues of UC patients, which is a compensatory response to slow down the development of UC. However, as the increasing level of *miR‐29a/b* expression was limited and not enough to prevent the disease progression, we employed a LMSH‐NPs‐miR‐29a/b hydrogel to deliver more *miR‐29a/b* for effective treatment (Figures 1K and 6L–M). Meantime, the heterozygous mice (*MiR‐29ab^+/−^*) didn't exhibit excessive inflammatory infiltration and T cell overactivation, further supporting our above viewpoint (Data was not displayed). To clarify the role of *miR‐29a/b*, we used *MiR‐29ab1*‐deleted mice to reveal that *miR‐29a/b* inhibited severe colitis by reducing the inflammatory response. More intriguingly, we discovered the major protective role of *miR‐29a/b* was mediated by the critical cell populations in mesenchyme rather than in epithelium. Furthermore, transcriptome analysis showed that *miR‐29a/b* suppressed inflammatory responses and inhibited excessive activation of CD8^+^ T cells through downregulating the *Ifng*‐JAK‐STAT signaling pathway. Our work also identified CD8^+^ T cell differentiation as important regulatory target in UC. Additionally, the accumulation of these two miRNAs in colorectal adenocarcinama is negatively correlated with T cell differentiation and the TNF‐α signaling pathway, indicating that *miR‐29a/b* may also participate in the occurrence and development of colorecal tumors.

Despite the fact that the participation of CD8^+^ T lymphocytes in colitis has been discussed and reported for a while [26], more thorough research should be carried out to determine the function and regulation of CD8^+^ T cells in the pathophysiology of UC. In this context, new researches sheds light by recognizing unique CD8^+^ T cell characteristics in IBD patients and grouping them according to RNA and protein expression patterns [8]. However, the transcriptional regulation underlying the differentiation towards these different populations is unknown. In the present work, we found that *miR‐29a/b* activated CD8^+^ T lymphocytes and promoted their differentiation into Tc1 and Tc17 cells by targeting *Ifng* at the mRNA levels. Our findings revealed that rIfng greatly increased the expression of p‐Stat1 and IL‐17 in CD8 cells but had no effect on the expression of p‐Stat3, showing that p‐Stat3 does not rely on Ifng to enhance CD8^+^ T cell differentiation. Additionally, the considerable drop in IL‐17 expression at protein level induced by JAK‐STAT inhibition demonstrated the relevance between STAT and IL‐17 in CD8^+^ T cells. Furthermore, many stromal cells, including CD8^+^ T cells, can release Ifng that activates the JAK‐STAT pathway to promote CD8^+^ T cell activation and differentiation into Tc1 and Tc2 cells. Tc1 cells then release the Ifng cytokine to further stimulate CD8^+^ T cell differentiation and form a positive feedback cycle. The overactivation of CD8^+^ T cells might be related to infiltration of immune cells, upregulated immune‐related pathways and enhanced innate and adaptive immune responses and a damaged intestinal barrier. A recent study found *miR‐29a* attenuated CD8^+^ T lymphocyte exhaustion and induced memory‐like CD8^+^ T lymphocytes during chronic infection [20]. Furthermore, *miR‐29a/b* pre‐programmes the immune response in naïve cells and improve the memory response in a viral infection model [19]. Interestingly, we firstly find that *miR‐29a/b* also act as switches that antagonize CD8^+^ T cell overactivation and differentiation in patients with UC. Taken together, CD8^+^ T cell populations exhibit high heterogenicity under different conditions, emphasizing the necessity of in‐depth research on CD8^+^ T cell function in patients with IBD. Of course, *miR‐29a/b* may participate in the regulation of T‐cell dysfunction through multiple signaling pathways or target genes. First, *miR‐29a/b* can target and inhibit the expression of genes related to co‐stimulatory molecules such as CD28, making it difficult for T cells to be fully activated and leading to T cell dysfunction [27]. Second, an increase in *miR‐29a/b* expression can suppress T‐bet expression, blocking Tc1 cell differentiation and affecting the normal function of T cells [27]. Third, *miR‐29a/b* may inhibit the expression of cyclin D1, blocking the progression of the T cell cycle and suppressing the T cell proliferation [28].

To identify the major target genes regulated in the DSS model, we made the intersection of the *miR‐29* target gene dataset, the gene set considerably elevated in *miR‐29* mutant mice, and the gene set significantly dysregulated in UC patients. We discovered that *IFNG* is the gene that fits the above criteria and has the most substantial alterations, and it was shown to be the most important target gene modulated by *miR‐29* in UC. Ifng, an important upstream regulatory factor, is thought to be one of the primary causes of this overreaction of the immune system, which leads to severe mucosal injury and extensive leukocyte infiltration [29]. It is essential for immunological responses in infection, particularly intracellular bacterial infections [30]. Interestingly, a recently published study found that activation‐induced downregulation of *miR‐29a/b* facilitated Ifng production in activated CD4^+^ T cells and natural killer (NK) cells to resist intracellular bacterial infection and that *miR‐29a/b* directly targeted *Ifng* [23]. At first glance, our findings appear contradictory as *miR‐29a/b* and its target *Ifng* may play distinct roles in bacterial infection and DSS‐induced colitis. In addition, *Ifng^−/−^* mice with reduced DSS‐induced inflammation demonstrated the critical function of this cytokine in the onset of colitis [31]. Furthermore, mice lacking *miR‐29a* displayed worsening colitis along with increased levels of IL‐23 and Th17 hallmark genes in the intestinal mucosa after being challenged with DSS [24]. These findings are concordant with our results, and our research expands the breadth of the immunoregulatory roles of *miR‐29a/b* in UC. Additionally, epithelial Ifng signaling orchestrates gut immunity via controlling the development of pathogenic CD4^+^ T cell responses [32], indicating the role of antigen presentation and cell‐autonomous defense mechanisms [33]. More importantly, our research proposes a mechanism by which stromal Ifng directly stimulates CD8^+^ T cells followed by excessive activation of inflammation and barrier disruption via the JAK‐STAT1 signal pathway. Furthermore, we also find Stat3 activates CD8^+^ T cells Ifng‐independently, and more precise mechanisms deserve further research in future.

Many cytokines are known to signal through the JAK‐STAT pathway. Interferons bind to surface receptors in this pathway, triggering a series of events that eventually cause STAT proteins to get phosphorylated intracellularly. Consequently, as a transcription factor, activated STAT results in sufficient immunological responses [34]. Furthermore, immune response dysfunction is associated with overactivation of the JAK‐STAT signal, and thus, newly discovered JAK inhibitors have been demonstrated to be successful in treating autoimmune conditions like UC [35] and rheumatoid arthritis [36]. Despite the development of many JAK inhibitors effective in UC, similar clinical responses were not duplicated in patients with CD with moderate‐to‐severe disease [35]. In addition, though increasing evidence indicates the roles of the various STATs in the cellular compartments of lymphocytes, macrophages and intestinal epithelial cells (IECs) [37], it is unclear whether epithelial or mesenchymal cells contribute significantly to the immune response. Therefore, our results further elucidate molecular mechanisms of JAK‐STATs contributing to UC and may provide new avenues for UC therapeutic development.

The biological processes responsible for intestinal mucosa injury and repair are distinct but interconnected. A fresh injury or the quick healing of injured tissues may be the cause of changes in the levels of *miR‐29a/b* and other variables seen in the damaged mucosa. Interestingly, recent research demonstrated that *miR‐29a/b* controlled intestinal epithelial regeneration and wound healing by reacting with circHIPK3 [38]. Furthermore, *miR‐29a/b* influences intestinal epithelial barrier function via interaction with the long noncoding RNA (lncRNA) UC.173 [3]. Recently, there is a proof now that lncRNAs represent a new class of master regulators of intestinal epithelial homeostasis and play an essential roles in the development, adaptability, and permeability of the gut mucosa [3, 4, 31]. It is well known that lncRNAs can carry out a variety of biological tasks by interacting with miRNAs as inhibitors or RNA decoys to decrease the availability and synthesis of miRNAs [6]. These findings provide a possibility for the potential of *miR‐29a/b* in protecting epithelial integrity and post‐injury epithelia recovery in patients with UC. However, the effectiveness and mechanism have not been fully understood and deserve further investigation in the future.

Altogether, *miR‐29a/b* may serve as a generalizable biomarker of immune response in UC, several limitations should be considered when applying the findings to the broader population.

On the one hand, colitis induced by DSS reproduces some of the key features of human UC, it has advanced our understanding of the disease and exhibited some translational value. While the complexity of clinical practice in UC patients cannot be entirely replicated by the DSS model. First, a major limitation is that the intricate pathophysiology of human UC, which is impacted by a number of environmental, genetic, and immunological variables, is not entirely replicated by DSS‐induced colitis [39]. Second, DSS‐induced colitis represents a T‐cell‐independent, chemically induced model, primarily driven by innate immune responses, showing that the DSS model is unable to replicate the UC patients' immunological microenvironment [40]. Third, we conducted acute colitis mouse model by treating the C57BL/6 mice with 3.5% DSS for 5 days which is different from the chronic colitis. The histological changes of chronic inflammation were often accompanied by intestinal fibrosis inducing by inciting inflammation and causing damage to the intestinal lining [41]. So, the model may be less effective at chronic enteritis and UC patients with fibrosis.

On the other hand, the study's limitations include its emphasis on gut cells isolated from mice and its evaluation of the effects of *miR‐29a/b* in specific loss‐of‐function experimental scenarios. Since inflammatory IL‐17 responses are not limited to the intestine, research into different types of pathological inflammation in other organs will be crucial to comprehending the wider effects of miR‐29a/b. Furthermore, our research surely overlooks many other pertinent targets in a wide range of cells in a variety of settings, as miRNAs usually target several gene transcripts. Therefore future validation will require expanding the other mouse strains or humans.

## 4 Conclusion

In summary, we show that *miR‐29a/b* is upregulated in patients with UC and in DSS‐induced colitis‐affected mice and that this upregulation relieves the development of significant inflammation in the colon. Thus, our findings suggest that *miR‐29a/b* regulates CD8^+^ T cell activation and differentiation towards Tc1 and Tc17 cells and a positive feedback mechanism in UC possibly via the *Ifng*‐JAK‐STAT signal. Therefore, our results therefore imply that the elevated expression of *miR‐29a/b* seen during inflammatory illness may reduce the severity of colitis, which may have therapeutic consequences. More significantly, food consumption allows plant‐based miRNAs to reach the human body's tissues and circulation. Additionally, once they are within the body, they will have biological impacts via controlling the expression of target genes, which will impact the body's physiological processes. Our study offers a theoretical foundation for creating dietary regimens for enteritis patients.

## 5 Material and Methods

### 5.1 Ethics Statement 

All experiments involving animals and human patients were conducted according to the ethical policies and procedures approved by the ethics committee of the Faculty of Pharmacy, Cairo University, Egypt (Approval no. AW31012202‐4‐2). Eight samples of human fresh colon colitis mucosa from patients with UC, along with adjacent normal tissue samples, were collected while the patients underwent a colonoscopy in The Chinese Academy of Medical Science Cancer Hospital and Beijing Hospital. Experimental approval (CAUHR2021020) was provided by the Human Research Ethics Committee of China Agricultural University. Each participant provided written informed consent. Tissue was immediately saved in 4% paraformaldehyde. Mice were housed in ventilated cages (maximally six mice per cage) in particular pathogen‐free circumstances. The mice were maintained on a standard 12:12 light cycle and had ad libitum access to autoclaved food and water. All trials, unless otherwise noted, used 8–12 week old littermate mice who were age and sex matched.

### 5.2 Animal Experiment

We looked at both male and female animals in our study, and the results are comparable for both sexes. The *MiR‐29ab1^−/−^* mice and Vil‐cre mice were kindly provided by Prof. Zheng‐quan Yu of China Agriculture University. Mice with an intestinal epithelial cell‐specific *miR‐29ab1* deletion *(VillinCre;MiR‐29ab1^flox/flox^*) were generated by crossing Vil‐cre mice^4^ with those harboring loxP‐flanked *MiR‐29ab1* (*MiR‐29ab1^flox/flox^*). Every mouse was produced using the C57BL/6 background. Mice were fed 3.5% (w/v) DSS (MP Biomedicals, Santa Ana, CA) of molecular weight 36,000–50,000 in water for 5 days, then three to 5 days of just drinking water in order to treat acute colitis. For statics treatment, statics acquired from MCE was injected intraperitoneally at 25 mg/kg per mouse 1 day before 3.5% DSS treatment and once a day during DSS administration. We observed the health condition of DSS‐treated mice and recorded body weight as well as the presence of diarrhea and bloody stools to score the severity of colitis. Mice were killed at the indicated points; colon length was measured and colon tissues were collected for further analysis. Hematoxylin and eosin (H&E) staining was used to grade the pathology blindly, taking into account factors such as depth of injury, crypt destruction, and inflammatory intensity, as previously reported^5^.

### 5.3 In situ Hybridization

The *miR‐29a/b* in situ hybridization assay was carried out on mice colon tissues as previously described but with modifications [6]. Briefly, digoxigenin‐labeled locked nucleic acid (LNA) probes purchased from Exiqon were used following the detailed procedure provided by the manufacturer's protocol. Hybridization of *miR‐29a/b*, scrambled LNA probes and the positive control U6 (Exiqon, Vedbaek, Denmark) were hybridized overnight at 61°C. Positive staining of *miR‐29a/b* was detected using an anti‐digoxigenin antibody (Roche, Basel, Switzerland) and BM purple substrate (Roche). The sections were incubated with the NBT/BCIP solution (Roche) for color response following several buffer washes. The mounted slides were visualized by fluorescence microscopy (Leica, Germany)

### 5.4 Cell Culture and Transfection

For in vitro experiments, 293T cells were cultured in DMEM supplemented with 10% fetal bovine serum (FBS, ThermoFisher) and 1% penicillin‐streptomycin. Transfecting 293T cells temporarily was accomplished with the Lipofectamine 2000 reagent (Invitrogen, Eugene, USA) with 40 nm of miR‐29a/b‐3p microRNA mimics (mimics‐ miR‐29a/b) or the negative control (mimics‐NC) or miR‐29a/b ‐3p microRNA inhibitor (inhibitor‐ miR‐29a/b) or the scrambled RNA (inhibitor‐NC) in compliance with the manufacturer's guidelines (Invitrogen, Carlsbad, CA).

The mouse CD8^+^ T cell line CTLL‐2 was grown in RPMI 1640 (Gibco, San Diego, CA) supplemented with 10 ng/mL IL‐2 (SinoBiological, USA). For miR‐29a/b overexpression or inhibition, cells were treated with 40 nm of the above microRNAs and performed according to the manufacturer's instruction using Neon transfection reagent (Thermo Scientific, Waltham, MA, USA) with a Neon electrotrotransfection system at 1700 V for 20 ms. The sequences used for cell transfection are located in Table S1, Supporting Information.

For IFN‐γ treatment, CTLL‐2 cells were cultured with 10 ng/mL IL‐2 and stimulated with 3.5 µg/mL CD3 (Biolegend, 100340) and 1 µg/mL CD28 (Biolegend, 102116) under the condition of supplementation with or without 200 ng/mL of recombinant mouse IFN‐γ (PreproTech, 315‐05) for 48 h. Next, CTLL‐2 cells were treated with 200 ng/mL of IFN‐γ under the condition of supplementation with or without 100 µm Ganoderic Acid A (MCE, HY‐N1447) for 48 h.

### 5.5 Dual Luciferase Reporter Assays

For target validation, the 3' UTR of the target mRNA was cloned into the pmirGLO vector just downstream of the Renilla luciferase gene, spanning at least 400 base pairs (bps) around the anticipated binding site of *miR‐29a/b*. (Jima, Suzhou, China). Nucleotides in the target site complementary to the *miR‐29a/b* seed region were altered in order to produce reporters with mutant 3' UTRs. (Jima, Suzhou, China). For normalization of transfection efficiency, 293T cells were cotransfected with 50 ng of the above vectors, mimics‐NC and mimics‐miR‐29a/b, as well as inhibitor‐NC and inhibitor‐miR‐29a/b. Using the dual‐specific luciferase assay kit (Promega, Madison, WI), luciferase assays were measured. At least three replications of each reporter assay were conducted. The sequences used for dual luciferase activity assays are located in Table S2, Supporting Information.

### 5.6 RT‐PCR

Total RNA was isolated from cells or colon tissues of mice in accordance with the manufacturer instructions and cDNA was generated with All‐In‐One RT Master Mix. Quantitative analysis of expression was performed using SYBR Premix Ex Taq (Themo Scientific). The relative gene expression was normalized to *Actb* (the gene encoding β‐actin). Using the Mir‐X miRNA First‐Strand Synthesis kit, 1 µg of total RNA was utilized to synthesize the cDNA for the miRNA RT‐PCR. (Takara Biotechnology, Shiga, Japan). The generated cDNA was then subjected to qRCR according to the instructions provided by the manufacturer. The primers used are located in Tables S3 and S4, Supporting Information.

### 5.7 RNA‐Sequencing

After being separated from mouse colon tissue, RNA was kept in TRIzol and the whole‐genome sequencing was executed by the Novogene company (Beijing, China). GSEA software was utilized to carry out gene analysis with differential expression based on the *p* value from the differential expression analysis.

### 5.8 Histologic Analysis and Immunofluorescence and Immunochemistry

For histological analysis, the colon tissue was washed with ice‐cold PBS, fixed with 4% formaldehyde solution and embedded in paraffin. The colon tissues were sectioned at 5 µm intervals and were deparaffinized by sequential washes of xylene and serial dilutions of ethanol and double‐distilled water (ddH_2_O). For histological analysis, sections were stained with H&E. Crypt abscess, erosion, ulcer, elevated chronic colitis in the lamina propria, submucosal inflammation, basal plasmacytosis and crypt distortion were scored on a 0–5 scale. For immunofluorescence and immunochemistry, sections were treated to heat‐mediated antigen retrieval in sodium citrate buffer, and then sections were incubated in 1% hydrogen peroxide to stop the endogenous peroxidase activity. After washing three times with PBS and blocking for 1 h with blocking solution (Solarbio Life Science, Beijing, China), after that, slices were left overnight at 4 ° C to be treated with primary antibodies, including for anti‐mouse IL‐1β (CST, 4970s), p65 (CST, 8242), CD45 (Santa Cruz, sc‐28369), F4/80 (Santa, sc‐52664,), Ki67 (Abcam, ab15580,), Pcna (Santa Cruz, sc‐56,). After incubation with the antibodies, sections were treated with the relevant secondary antibodies after three PBS washes followed by reacting with 3, 3'‐diaminobenzidine and covering sections with neutral balsam. Alternatively, following secondary antibody incubation, slices were coated with a quenching agent and stained for 5 min.

### 5.9 Western Blotting and Antibodies

Tissues or cells were subjected to WB analysis according to standard protocols and lysed in RIPA buffer that has been enhanced with inhibitors of the protease and phosphatase (Beyotime Biotechnology, Beijing, China). A BCA protein assay kit was used to measure the quantities of proteins, (Beyotime Biotechnology, Beijing, China), 20 µg of total protein was separated by 6%‐12% sodium dodecyl sulfate (SDS)‐PAGE and transferred to polyvinylidene difluoride membranes (Millipore, Billerica, MA). After blocking the membranes with 5% nonfat dry milk, they were incubated at room temperature for 1 h and then at 4 ° C for an overnight incubation period with the primary antibody. The following antibodies were used: IL‐1β (CST, 4970s), p‐p65 (CST, 3033T), p65 (CST, 8242), TGF‐β (Abcam, ab64715), β‐actin (CST, 8457T), CyclinD1 (Abcam, ab134175), Occludin (Abcam, ab216327), ZO‐1 (Santa, sc‐33725,), E‐cadherin (CST, 3195T), Claudin1 (sc‐166338), Pcna (sc‐56), Stat3 (Abcam, ab76315,), IL‐17 (sc‐374218), Ifng (sc‐8423), Ifitm3 (sc‐100768), p‐Stat3 (sc‐8059), p‐Stat1 (sc‐136229), Stat1 (sc‐136229). The suitable secondary antibodies were incubated with the membranes and the resulting protein staining was visualized using an electrochemiluminescence kit (Millipore).

### 5.10 EdU Staining

For EdU incorporation assay, 10 mm Edu dissolved in ddH_2_O was i.p. injected into mice, and they were sacrificed 2, 24, 48 h following the injection. Then, the colon tissues were preserved with 4% formaldehyde solution, embedded in paraffin, sectioned at 5 µm and deparaffinized. The sections were stained for EdU using the BeyoClick EdU Cell Proliferation Kit (Beyotime Biotechnology, Beijing, China), in line with the manufacturer's guidelines.

### 5.11 Isolation of Intestinal Epithelium and Mesenchymal Cells

After longitudinal dissection, mouse colons were washed three times with ice‐cold PBS buffer and sliced into 5‐mm‐long pieces. These pieces were then incubated for 30 min at 37 ° C with continuous shaking at 200 rpm in PBS buffer adding 5 mm EDTA in order to release the epithelial cells. Suspended cells were collected after vigorous shaking, the remaining colon tissue pieces were digested with 10 mL digestion solution containing 300 U/mL collagenase II (Thermo Fisher, Waltham, MA) and 0.08 U/mL dispase II (Beyotime Biotechnology, Beijing, China) for another 30 min incubation at 37 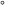 C on a rotating platform. After digestion, epithelial cells as well as mesenchymal cells were shaken vigorously and collected into a new tube after being passed through 100 µm cell strainers, then were pelleted at 300 × *g* for 5 min. Single‐cell suspensions were produced after treatment with dispase II (Beyotime Biotechnology, Beijing, China) and DNase I (Beyotime Biotechnology, Beijing, China), then passing the mixture through a 40 µm cell strainer.

### 5.12 Flow Cytometric and Cell Sorting

BD FACS Arial 3.0 was used to analyze or sort single‐cell suspensions in accordance with accepted practices. The resulting fcs files were imported and analyzed with FlowJo V10.

For membrane protein staining, single‐cell suspensions were stained with DAPI (Beyotime Biotechnology, Beijing, China) used as a viability dye to remove dead cells followed by fluorophore‐conjugated antibodies for 15 min on ice. The following antibodies were used: APC/Cyanine7 anti‐mouse CD326 (Biolegend, 118217), PerCP/Cyanine 5.5 anti‐mouse CD45 (Biolegend,103131), PE/Cyanine7 anti‐mouse CD19 (Biolegend, 115519), PE anti‐mouse CD3 (TONBO, 50‐0032‐U100), APC anti‐mouse CD8 (Biolegend,100711), and FITC anti‐mouse CD4 (Biolegend, 100406).

For cytokine staining, single‐cell suspensions underwent Zombie staining (Biolegend, 23101) used as a viability dye to remove dead cells followed by fixation, membrane permeabilization (Biolegend, 426803) and fluorophore‐conjugated antibodies for 15 min on ice. The following antibodies were used: FITC anti‐mouse Ifng (eBioscience, 11‐7311‐81), PE/Cyanine7 anti‐mouse Granzyme B (Iivitrogen, 25‐8898‐82) and APC/Cyanine7 anti‐mouse IL‐17(Biolegend, 506921).

### 5.13 Statistical Analysis

Every experiment that was presented was carried out at least three separate occasions. Results were all presented as mean ± SD and the survival data were presented by Kaplan–Meier plot. Data analysis was carried out with SPSS (IBM), Excel (Microsoft) and GraphPad Prism (GraphPad 397 Software). Image J (NIH) was used for blot and image analysis. FlowJo 10.4 (FlowJo 398 LLC.) was used for flow cytometry analysis. GSEA 4.1.0 (Broad Institute) was used for GSEA. The significance of the differences between two groups was analyzed by Student's *t*‐test. Non‐significant was represented by ns. For multiple groups, the significance was analyzed with Duncan's post hoc test following one‐way ANOVA. Significant differences were established at the level of *p < 0.05, **p < 0.01, ***p < 0.001. Animals were randomly assigned to groups based on genotype. The animal experiments were not carried out in a blinded fashion.

## Conflicts of Interest

The authors declare no conflicts of interest.

## Supporting information




**Supplementary File 1**: exp270058‐sup‐0001‐figureS1‐S7.docx.

**Supplementary File 2**: exp270058‐sup‐0002‐tableS1‐S4.docx.

## Data Availability

The Gene Expression Omnibus has been deposited with the RNA‐seq data presented in this study (GSE253734). One of the samples from WT mice and another sample from *MiR‐29ab1^−/−^* mice were excluded from analysis.
