# Supplementary material for: MiR‐29a/b Suppresses CD8+ T Cell Effector Function and Intestinal Inflammation
Source: Exploration (Beijing). 2025 Jun 10;5(4):20240363. doi: 10.1002/EXP.20240363 (PMC12380058; doi:10.1002/EXP.20240363)
Supplement: Supplementary file 1 — exp270058‐sup‐0001‐figureS1‐S7.docx. [file EXP2-5-20240363-s001.docx]

**_­­
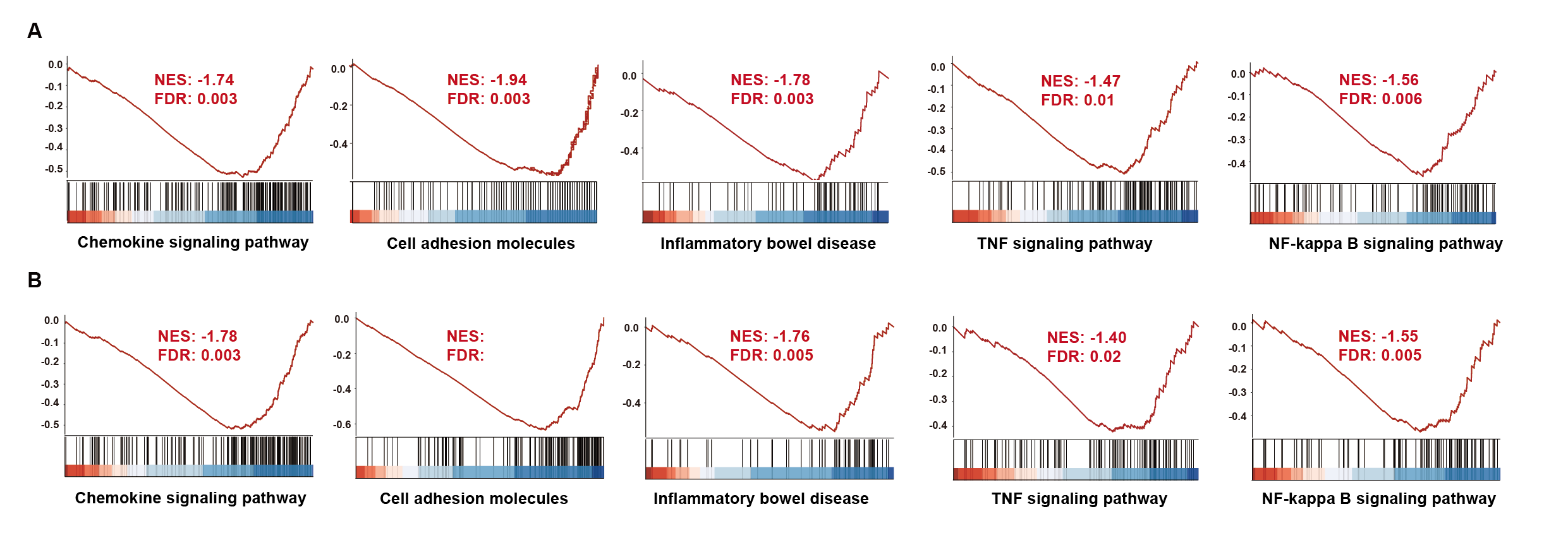
_**

**Supplementary Data Fig. S1.** *MiR-29a/b* **is closely related to the regulation of T cell fate and inflammation signaling pathway.** A, Immune-related pathways enriched in GSEA on genes related to hsa-miR-29a-3p in COAD and READ data set from TCGA platform. n = 8 normal tissues and 457 tumors in COAD, 3 normal tissues and 162 tumors in READ. B, Immune-related pathways enriched in GSEA on genes related to hsa-miR-29b-3p in COAD and READ data set from TCGA platform. n = 8 normal tissues and 457 tumors in COAD, 3 normal tissues and 162 tumors in READ.


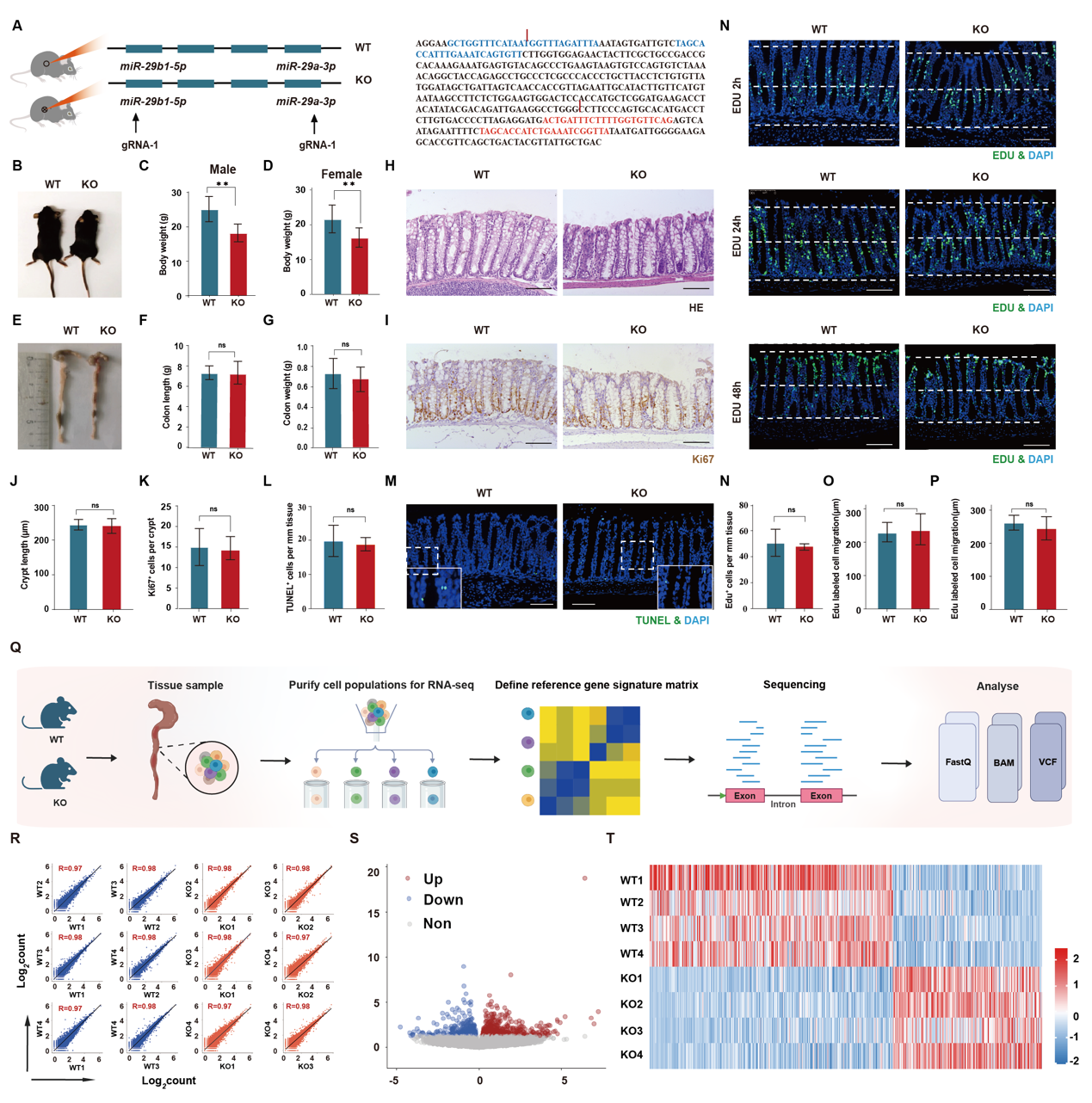


**Supplementary Data Fig. S2. The phenotypes of *MiR29ab1^-/-^* mice in physiological status.** A, Schematic diagram showing construction strategy for *MiR29ab1^-/-^* mice. B, Photos of WT and *MiR29ab1^-/-^* mice. C-D, Body weight of WT and *MiR29ab1^-/-^* mice. n = 18 mice in each group. E, Photos of colon from WT and *MiR29ab1^-/-^* mice. F-G, Quantification of the length and weight of colon from WT and *MiR29ab1^-/-^* mice. n = 18 mice in each group. H, Histological images of colon from WT and *MiR29ab1^-/-^* mice. Scale bar: 100 µm. n = 6 mice in each group. I, Immunohistochemistry for Ki67 in the colon from WT and *MiR29ab1^-/-^* mice littermates. n = 3 mice in each group. J-L, Quantification of crypt length, Ki67^+^ cell and TUNNEL^+^ cell in colon of WT and *MiR29ab1^-/-^* mice. n = 3-6 mice in each group. M, Representative images of TUNEL in the colon from WT and *MiR29ab1^-/-^* mice littermates. n = 6 mice in each group. N-P, The rate and quantification of EDU^+^ cell migration in colon of WT and *MiR29ab1^-/-^* mice. Q, Schematic diagram showing the process of bulk RNA-sequencing on colon tissues from WT and *MiR29ab1*^-/-^ mice in physiological conditions. R, Correlation between data from WT mice or *MiR29ab1*^-/-^ mice analyzed by spearman method. n = 4 mice in each group. S, Volcano plot of genes detected in the RNA-sequencing. n = 4 mice in each group. T, Heatmap of differential gene expression analysis on colon tissues from WT and *MiR29ab1*^-/-^ mice. The cutoff was *P* < 0.05. n = 4 mice in each group. Data are presented as mean ± SD. Student's *t*-test. * *P* < 0.05, ** *P* < 0.01.


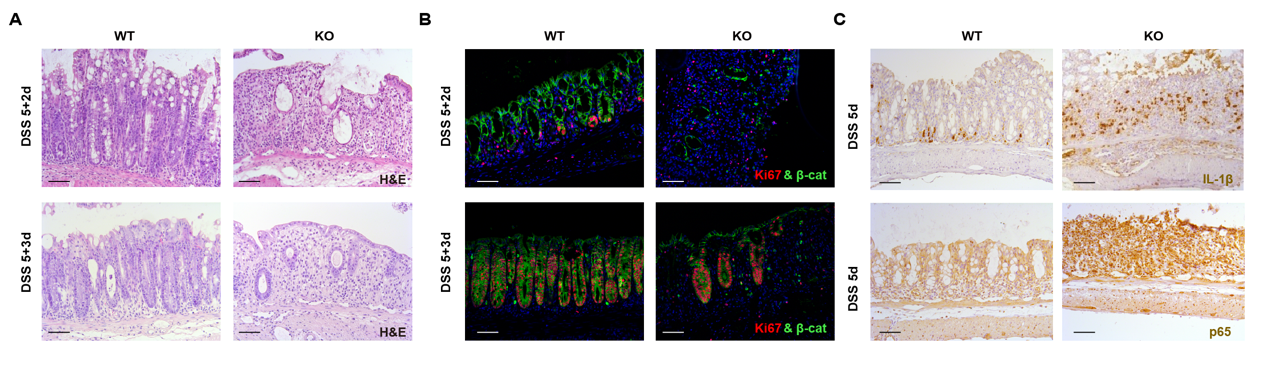


**Supplementary Data Fig. S3. Deletion of *MiR-29ab1* increases the severity of DSS-induced colitis and colon innjury.** A, Histological images of colon from WT and *MiR29ab1^-/-^* littermates following 5+2 and 5+3 days of DSS treatment. Scale bar: 100 µm. n = 2-3 mice in each group. B, Double immunofluorescence for Ki67 as well as β-catenin in the colon from WT and *MiR29ab1^-/-^* mice following 5+2 and 5+3 days of DSS treatment. n = 2-3 mice in each group. C, Immunohistochemistry for IL-1β and p65 in the colon from WT and *MiR29ab1^-/-^* mice littermates following 5 days of DSS treatment. n = 3 mice in each group. Data are presented as mean ± SD. Student's *t*-test. * *P* < 0.05, ** *P* < 0.01.


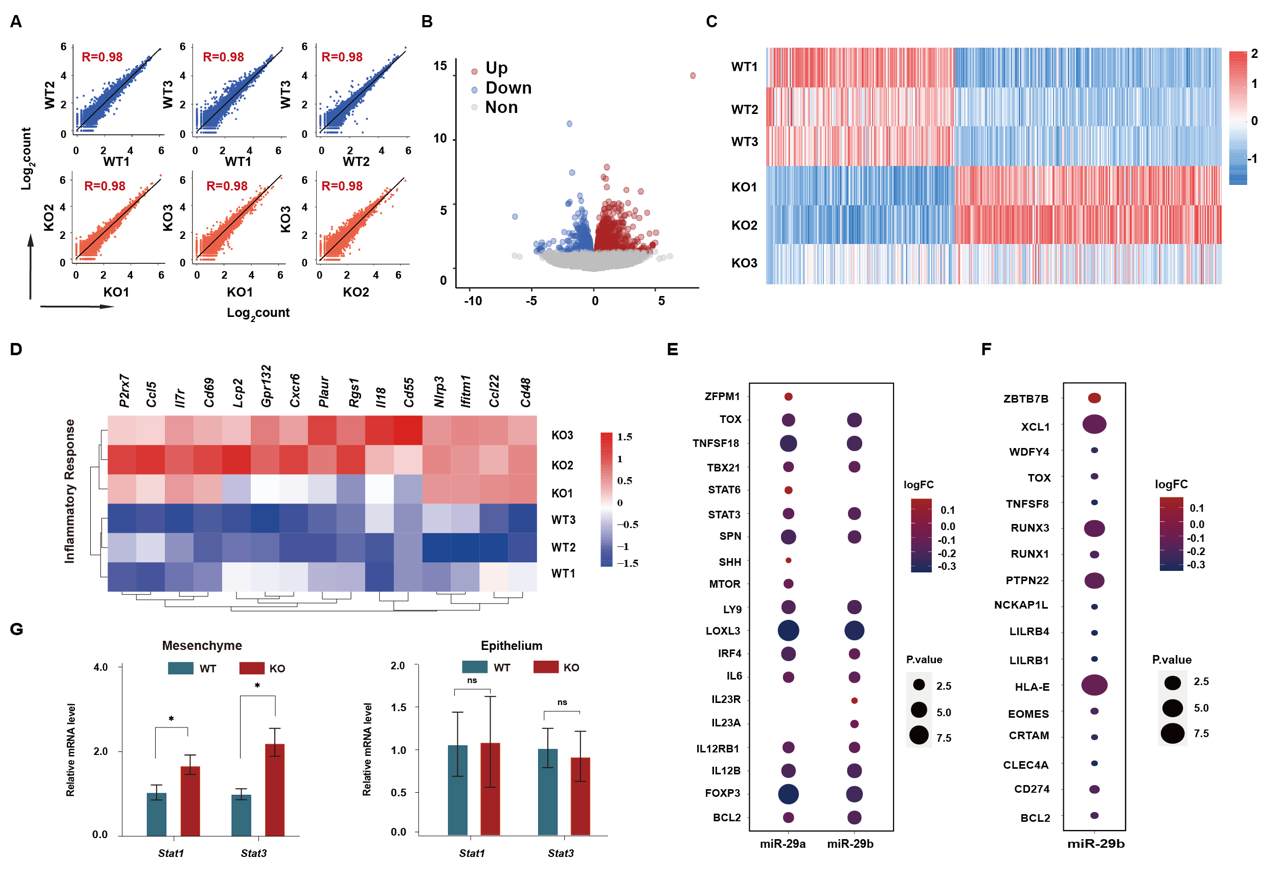


**Supplementary Data Fig. S4. Deletion of *MiR-29ab1* regulates T cell activation and differentiation in colonic stromal cells.** A, Correlation between data from WT mice or *MiR29ab1^-/-^* mice analyzed by spearman method. n = 3 mice in each group. B, Volcano plot of genes detected in the RNA-sequencing. n = 3 mice in each group. C, Heatmap of differential gene expression analysis of colonic tissues from WT and *MiR29ab1^-/-^* mice. The cutoff was *P* < 0.05. n = 3 mice in each group. D, Heatmap of differential gene expression analysis on colon tissues from WT and *MiR29ab1*^-/-^ mice related to inflammation response. The cutoff was *P* < 0.05. n = 3 mice in each group. E, Genes participated in differentiation of CD4 or CD8 league correlated to hsa-miR-29a-3p/ hsa-miR-29b-3p. F, Genes participated in activation of CD8^+^ T cells correlated to hsa-miR-29b-3p. G, qPCR analysis for *Stat1* and *Stat3* in the mesenchyme and epithelium of colon, respectively. n = 6 mice in each group. Data are presented as mean ± SD. Student's *t*-test. * *P* < 0.05, ** *P* < 0.01.


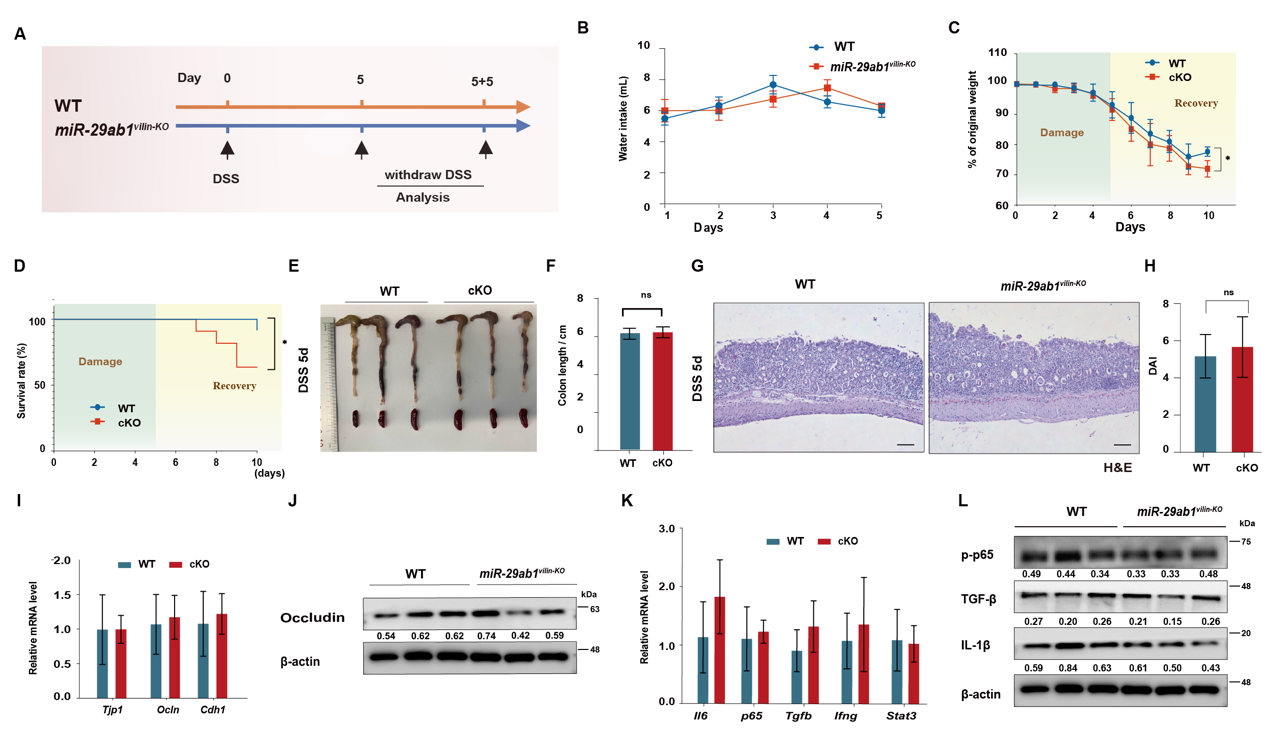


**Supplementary Data Fig. S5.** A, Schematic diagram showing DSS and analysis schedule. B, Quantification of water intake in *Vilin-Cre; MiR29ab1 ^+/+^* and *Vilin-Cre; MiR29ab1 ^fl/fl^* littermates. n = 6-8 mice in each group. C, Quantification of weight loss in *Vilin-Cre; MiR29ab1 ^+/+^* and *Vilin-Cre; MiR29ab1 ^fl/fl^* littermates following 5 days of DSS treatment. n = 6-8 mice in each group. D, Survival curve of *Vilin-Cre; MiR29ab1 ^+/+^* and *Vilin-Cre; MiR29ab1 ^fl/fl^* littermates after DSS treatment. n = 6-8 mice in each group. E-F, Histological images of colon and quantification of colon length from *Vilin-Cre; MiR29ab1^+/+^* and *Vilin-Cre; MiR29ab1 ^fl/fl^* littermates following 5 days of DSS treatment. n = 6-8 mice in each group. G-H, Histological images of colonic tissue and quantification of DAI from *Vilin-Cre; MiR29ab1^+/+^* and *Vilin-Cre; MiR29ab1^fl/fl^* littermates following 5 days of DSS treatment. Scale bar: 100 µm. n = 6 mice in each group. I, qPCR analysis for *Tjp1*, *Ocln* and *Cldn1* of colon from *Vilin-Cre; MiR29ab1^+/+^* and *Vilin-Cre; MiR29ab1 ^fl/fl^* littermates following 5 days post-DSS. n = 6 mice in each group. J, Western blotting for Occludin from *Vilin-Cre; MiR29ab1^+/+^* and *Vilin-Cre; MiR29ab1 ^fl/fl^* littermates following 5 days post-DSS. β-actin was used as a loading control. n = 3 mice in each group. K, qPCR analysis for *Il6*, *p65, Tgfb, Ifng and Stat3* of colon from *Vilin-Cre; MiR29ab1^+/+^* and *Vilin-Cre; MiR29ab1 ^fl/fl^* littermates following 5 days post DSS. n = 6 mice in each group. L, Western blotting for p-p65, TGF-β and IL-1β from *Vilin-Cre; MiR29ab1^+/+^* and *Vilin-Cre; MiR29ab1^fl/fl^* littermates following 5 days post DSS. β-actin was used as a loading control. n = 3 mice in each group. Data are presented as mean ± SD. Student's *t*-test. * *P* < 0.05, ** *P* < 0.01.

­­
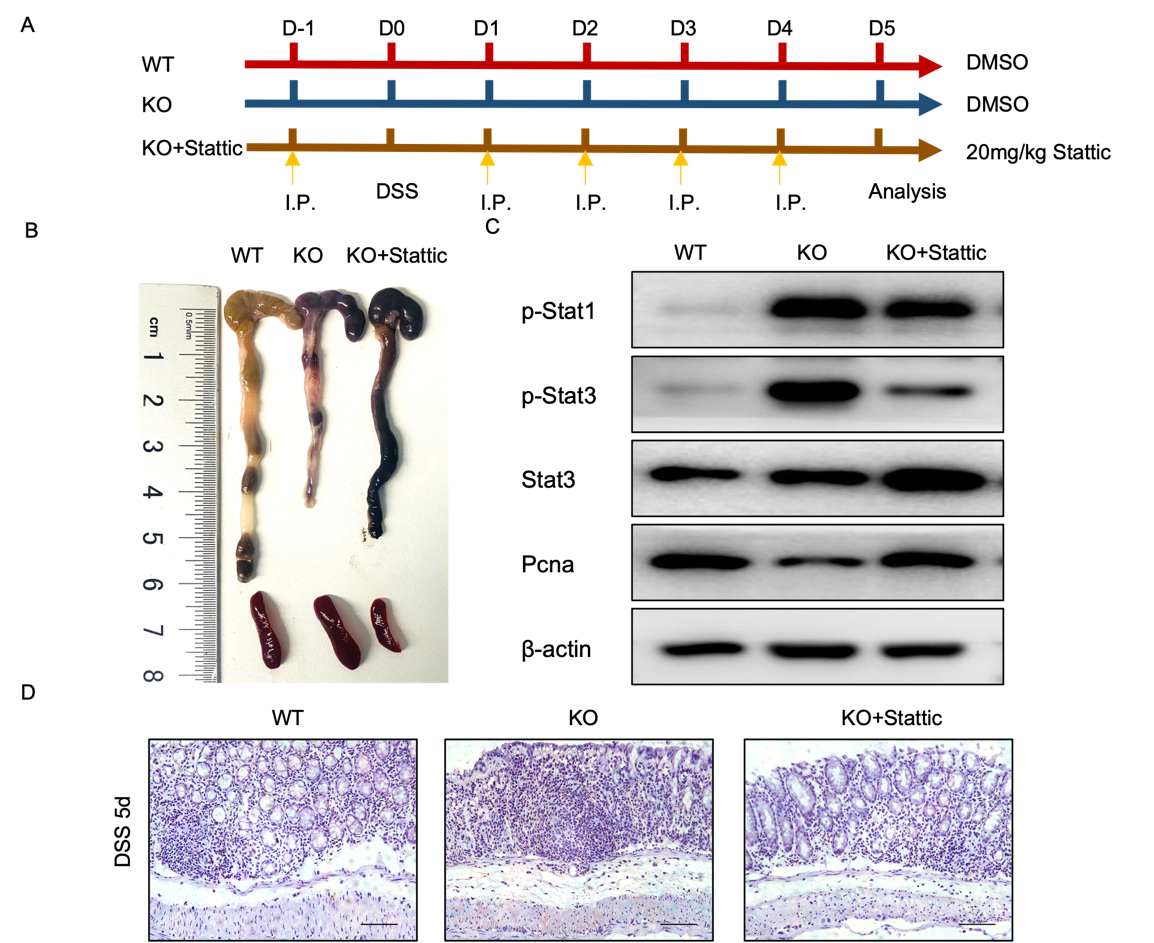


**Supplementary Data Fig. S6.** A, Schematic diagram showing DSS and analysis schedule. B, Histological images of colon following 5 days of DSS treatment. C, Western blotting for p-Stat1, p-Stat3, Stat3, and Pcna from *MiR29ab^+/+^*, *MiR29ab^-/-^* and *MiR29ab^-/-^* mice treated with Stattic following 5 days post DSS. β-actin was used as a loading control. n = 2-3 mice in each group. D, Histological images of colonic tissue from *MiR29ab^+/+^*, *MiR29ab^-/-^* and *MiR29ab^-/-^* mice treated with Stattic following 5 days post DSS. Scale bar: 50 µm. n = 2-3 mice in each group.


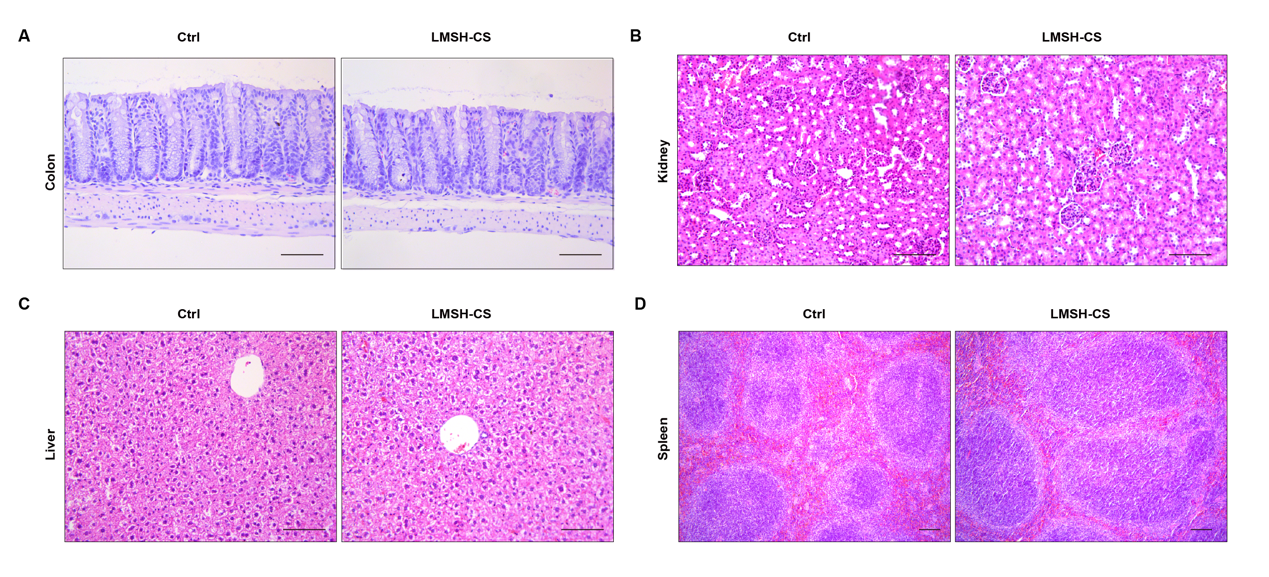


**Supplementary Data Fig. S7. LMSH-CS hydrogel exhibits no toxicity.** A-D, Histology for colon, kidney, liver and spleen from control and LMSH-CS hydrogel treated mice. Scale bar: 50 μm. n = 6 mice in each group. Data are presented as mean ± SD. Student's *t*-test. * *P* < 0.05, ** *P* < 0.01.
