# Supplementary material for: MiR‐29a/b Suppresses CD8+ T Cell Effector Function and Intestinal Inflammation
Source: Exploration (Beijing). 2025 Jun 10;5(4):20240363. doi: 10.1002/EXP.20240363 (PMC12380058; doi:10.1002/EXP.20240363)
Supplement: Supplementary file 2 — exp270058‐sup‐0002‐tableS1‐S4.docx. [file EXP2-5-20240363-s002.docx]

**Supplementary Table 1. Sequences for miR-29.**

| Primer name | Sequence (5' -3') |
| --- | --- |
| mimics-NC | 5'-UUGUACUACACAAAAGUACUG-3'  3'-CACUGAUUUCAAAUGGUGCUAUU-5' |
| mimics-miR-29 | 5'- UAGCACCAUUUGAAAUCAGUGUU -3'  3'- CACUGAUUUCAAAUGGUGCUAUU -5' |
| inhibitor-NC | 5'-CAGUACUUUUGUGUAGUACAA-3' |
| inhibitor-miR-29 | 5'-UAACCGAUUUCAGAUGGUGCUA-3' |

**Supplementary Table 2. Sequences used for dual luciferase activity assays.**

| Carrier name | Sequences (5'-3) |
| --- | --- |
| GP-miRGLO-mmu-Lincpint-*miR-29*-WT | ACATACATATATATATATACACATTTAAGGCATGCAAACGAGCAACTGCAGAGGCGTCTACAGTACTTAGCGTGTAGCAGGAACATCCAGGCTCTCCAGGCAGTTCACGGGTGCTTACACAGCCTGACGAGGTGCTCGCCGTGCCGCCATCCTTTCCTTGAGCGAACTGTACCCTGGGTTATGAACCTTGTTTGCCTGATAGTTCAG |
| GP-miRGLO-mmu-Lincpint-*miR-29*-Mut | ACATACATATATATATATACACATTTAAGGCATGCAAACGAGCAACTGCAGAGGCGTCTACAGTACTTAGCGTGTAGCAGGAACATCCAGGCTCTCCAGGCAGTTCACGCCACGATACACAGCCTGACGACCACGACGCCGTGCCGCCATCCTTTCCTTGAGCGAACTGTACCCTGGGTTATGAACCTTGTTTGCCTGATAGTTCAG |

**Supplementary Table 3. Primers for Quantitative Reverse-Transcriptase Polymerase Chain Reaction in this study.**

| Gene | Forward primer 5'-3' | Reverse primer 5'-3' |
| --- | --- | --- |
| *M-Ocln* | TTGAAAGTCCACCTCCTTACAGA | CCGGATAAAAAGAGTACGCTGG |
| *M-Tjp1* | GCCGCTAAGAGCACAGCAA | TCCCCACTCTGAAAATGAGGA |
| *M-Stat3* | CAATACCATTGACCTGCCGAT | GAGCGACTCAAACTGCCCT |
| *M-Akr1b8* | GACCAAGGCAGAATCCTCACC | AGATGCCCTTCGAGTGACAGT |
| *M-Lincpint* | GGAGGGTCCTTGCCTCACT | TGATCTGTGGTCATGTACTCCTG |
| *M-Chga* | ATCCTCTCTATCCTGCGACAC | GGGCTCTGGTTCTCAAACACT |
| *M-Clu* | AGCAGGAGGTCTCTGACAATG | GGCTTCCTCTAAACTGTTGAGC |
| *M-Anxa1* | ATGTATCCTCGGATGTTGCTGC | TGAGCATTGGTCCTCTTGGTA |
| *M-Cdh1* | CAGGTCTCCTCATGGCTTTGC | CTTCCGAAAAGAAGGCTGTCC |
| *M-Chgb* | GCTCAGCTCCAGTGGATAACA | CAGGGGTGATCGTTGGAACAC |
| *M-Mov10* | GAGGTTCGAGAGTTTTCTGGC | GCGATCTTCATTCCATACAGCAT |
| *M-Meis3* | CTTCTGTCACCGCTACATCAC | CTACCATCCCGATCCTCAATCA |
| *M-P2rx7* | GACAAACAAAGTCACCCGGAT | CGCTCACCAAAGCAAAGCTAAT |
| *M-Sult1d1* | ATGTCTTCAGGAGGGAGTTAGTG | CATCAGGCCGGGCTTCAAA |
| *M-Ugt8a* | ACTCCATATTTCATGCTCCTGTG | AGGCCGATGCTAGTGTCTTGA |
| *M-Pcna* | TTTGAGGCACGCCTGATCC | GGAGACGTGAGACGAGTCCAT |
| *M-Ccnd1* | GCGTACCCTGACACCAATCTC | CTCCTCTTCGCACTTCTGCTC |
| *M-Ascl2* | AAGCACACCTTGACTGGTACG | AAGTGGACGTTTGCACCTTCA |
| *M-Sca1* | AGGAGGCAGCAGTTATTGTGG | CGTTGACCTTAGTACCCAGGA |
| *M-Il6* | AGATACCACCAAGACCCACC | GGTGACCAGGGAGATTCGAA |
| *M-Ifng* | ATGAACGCTACACACTGCATC | CCATCCTTTTGCCAGTTCCTC |
| *M-p65* | AGGCTTCTGGGCCTTATGTG | TGCTTCTCTCGCCAGGAATAC |
| *M-Tgfb* | CTCCCGTGGCTTCTAGTGC | GCCTTAGTTTGGACAGGATCTG |
| *M-Gapdh* | GTGCCGCCTGGAGAAACCT | AAGTCGCAGGAGACAACC |
| *M-Cldn1* | GGGGACAACATCGTGACCG | AGGAGTCGAAGACTTTGCACT |
| *M-Il1b* | TGCACTACAGGCTCCG | TGCCGTCTTTCATTACAC |
| *M-Tnfa* | CCCTCACACTCAGATCATCTTCT | GCTACGACGTGGGCTACAG |
| *M-Stat1* | CCTGCGTGCAGTGATCGTTT | TCCAAATGCTTCCGTTCCCA |
| *M-Cxcl1* | CTGGGATTCACCTCAAGAACATC | CAGGGTCAAGGCAAGCCTC |
| *M-Cxcl2* | CCAACCACCAGGCTACAGG | GCGTCACACTCAAGCTCTG |
| *M-Cxcl3* | AACCGAAGTCATAGCCACAC | ACTTCTCTCCTGTCAGTTGGTG |
| *H-Lincpint* | GGCTGAGCTAGGTTTGCCAT | CAAGAGGTAGCTGGCGGAAA |
| *H-Akr1b8* | GTGACACCAGCACGCATTG | GCATTGAAGGGATAGTCTTCCAA |
| *H-Gapdh* | GGAGCGAGATCCCTCCAAAAT | GGCTGTTGTCATACTTCTCATGG |

**Supplementary Table 4. Sequences of the RT and RT-PCR primers for *miR-29a/b*.**

| Primer name | Sequence (5' -3') |
| --- | --- |
| miR-29a RT | CTCAACTGGTGTCGTGGAGTCGGCAATTCAGTTGAGTAACCGAT |
| miR-29b RT | CTCAACTGGTGTCGTGGAGTCGGCAATTCAGTTGAGAACACTGA |
| U6-RT | CGCTTCACGAATTTGCGTGTCAT |
| RT-miR-29a-F | TCACGTAGCACCATCTGAA |
| RT-miR-29b-F | TCACGTAGCACCATTTGAAA |
| RT-miR-29a/b-R | GTCAGTCCGTTTGGCCAGTA |
| RT-U6-F | CTCGCTTCGGCAGCACA |
| RT-U6-R | AACGCTTCACGAATTTGCGT |
